# PROTAC-Mediated Ternary Complex Stability with Ricin Toxin A: A Computational Perspective

**DOI:** 10.1021/acsomega.5c10223

**Published:** 2026-02-09

**Authors:** Fernanda D. Botelho, Salim T. Islam, Steven R. LaPlante, Tanos C. C. Franca

**Affiliations:** † Laboratory of Molecular Modeling Applied to the Chemical and Biological Defense (LMCBD), Military Institute of Engineering, Rio de Janeiro, Rio de Janeiro 22290-270, Brazil; ‡ Institut National de la Recherche Scientifique (INRS), Centre Armand-Frappier Santé Biotechnologie, 14850 Université du Québec, Institut Pasteur International Network, Laval, QC H7V 1B7, Canada; § PROTEO, the Quebec Network for Research on Protein Function, Engineering, and Applications, Universite Laval, Quebec, QC G1V 0A6, Canada; ∥ Center for Basic and Applied Research, Faculty of Informatics and Management, University of Hradec Kralove, Rokitanskeho 62, Hradec Kralové 50003, Czech Republic

## Abstract

Ricin is a potent toxin present in the seeds of the castor plant (*Ricinus communis*), which is widely distributed in tropical regions. To date, there are no approved antidotes or vaccines against ricin poisoning. Reported inhibitors have not yet achieved sufficient affinity, and vaccine candidates have shown limited efficacy, highlighting the need to explore alternative strategies for RTA neutralization. In this work, we performed a computational study to investigate the potential of using PROTACs (proteolysis-targeting chimeras) to induce the ubiquitination and subsequent proteasomal degradation of ricin. Specifically, we assessed the stability of RTA, the catalytic subunit of ricin, in complex with the E3 ligases VHL and CRBN, both widely employed in the PROTAC design. Several PROTAC candidates with distinct linkers were evaluated to identify linkers with greater potential to mediate the stable ternary complex formation between RTA and the ligases. Molecular docking and molecular dynamics simulations revealed three promising PROTACs, one targeting CRBN and two targeting VHL, as potential candidates for further *in vitro* validation. Overall, this study introduces PROTAC-mediated degradation as a novel and unexplored therapeutic strategy against ricin intoxication, laying the groundwork for future experimental investigations.

## Graphical abstract

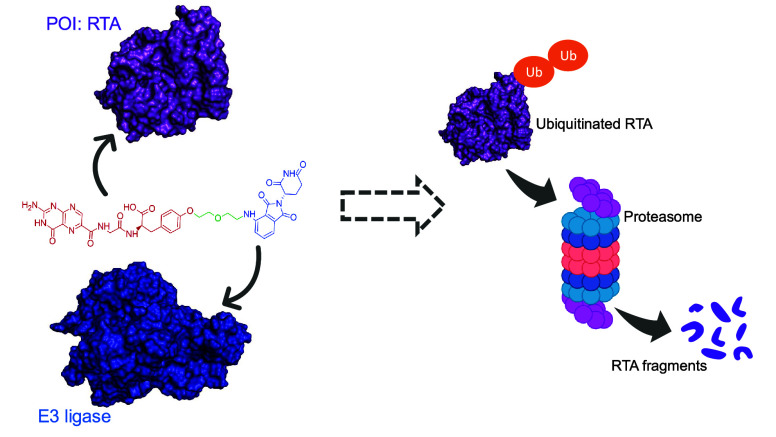

## Introduction

The discovery and development of new drugs is an undeniably complex process, with constant challenges and setbacks that may hinder the success of a given strategy in delivering effective medicines to the population. Traditionally, small molecules acting as competitive protein inhibitors have been widely used and frequently subjected to computational and experimental screening to assess the potential efficacy against various targets. However, their prolonged use can lead to drug resistance through mutations in target proteins, reducing therapeutic effectiveness. Moreover, many undruggable proteins require alternative therapeutic approaches beyond conventional methods.^1,2^

A promising alternative involves targeting protein–protein interactions (PPIs), which play key roles in intracellular signaling. Among such strategies, proteolysis targeting chimeras (PROTACs) have emerged as an innovative approach since their first reports in the early 2000s.^1–5^ PROTACs are small molecules composed of three modules: one end binds the protein of interest (POI) to be degraded, the other binds an E3 ubiquitin ligase (the “anchor”), and a linker connects these two binding moieties (Figure 1).^3^ By simultaneously binding both the POI and the E3 ligase, PROTACs trigger the POI ubiquitination and its subsequent degradation by the proteasome in a cycle that also involves the proteins E1 and E2 ligase.^4,6,7^ Unlike traditional competitive or allosteric enzyme inhibitors, which act in a 1:1 ratio with the target, PROTACs are catalytically recycled, enabling iterative action at lower doses. Furthermore, complete removal of the target protein abolishes not only its catalytic activity but also any noncatalytic functions.^2,3,8^

**Figure 1:**
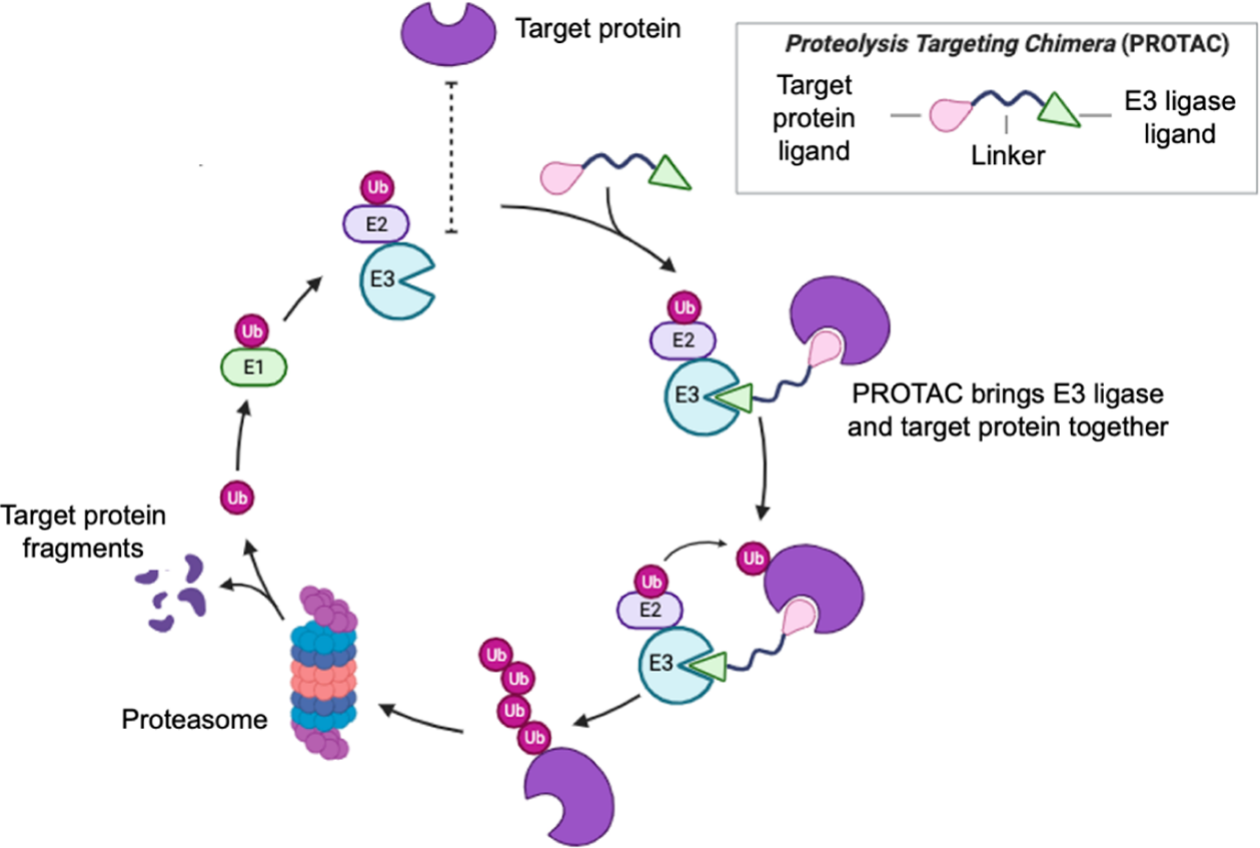
Cycle of the PROTAC-mediated ubiquitination of a target protein. The PROTAC acts as a molecular bridge, bringing the target protein (purple) close to the E3 ligase (blue). This induced proximity allows the E2 ligase to transfer ubiquitin molecules to the target, tagging it for recognition and subsequent degradation by the proteasome.

To date, no PROTAC-based drugs have been approved for clinical use, although several candidates are currently in different phases of clinical trials. Most PROTACs developed so far have been designed as anticancer agents, targeting specific proteins in cancer cells.^8–12^ However, in principle, these molecules can be tailored to degrade a wide variety of proteins, including exogenous ones. In this context, we computationally investigated the stability of ternary complexes formed by an E3 ligase, a PROTAC, and ricin toxin A (RTA), the catalytic subunit of ricin. Nonetheless, computational approaches are already widely applied in the field of PROTAC design, and studies have also demonstrated that molecular modeling can reliably reproduce experimental findings.^13–15^

Ricin is a highly potent toxin derived from the seeds of the castor bean plant, a species widely distributed in tropical regions. It is classified as a chemical weapon under the Chemical Weapons Convention^16^ (https://www.opcw.org/chemical-weapons-convention) due to its ease of extraction, high toxicity, water solubility, and other hazardous properties.^16^ Despite extensive efforts to develop antidotes for ricin poisoning, no specific or commercially available treatment exists, and management remains purely symptomatic.^17–19^ Ricin structure, mode of action, and other aspects have already been widely reviewed in the literature.^17–22^

To address the challenge of developing an antidote against ricin, multiple strategies have been pursued. Both computational and experimental studies have contributed to advances in the design of competitive and allosteric RTA inhibitors, ricin vaccines, and monoclonal antibodies capable of neutralizing the toxin in the human body.^17,20,22–26^ However, to the best of our knowledge, the use of a PROTAC to promote intracellular degradation of RTA has not yet been reported.

To explore this possibility, we conducted a computational study employing the conformational searching and scoring protein-linker-protein tool^27,28^ implemented within MOE (https://www.chemcomp.com/Products.htm) followed by molecular dynamics (MD) simulations to evaluate the feasibility of targeting RTA for degradation via the proteasome. In the case of ricin, such *in silico* methods are particularly valuable, as they help identify the most promising candidates for *in vitro* testing, while minimizing the risks inherent to handling this highly toxic substance in the laboratory.

## Methods

### Protein Preparation

The three-dimensional structure of RTA used in this study was the one complexed with N2-(2-amino-4-oxo-3,4-dihydropteridine-7-carbonyl)­glycyl-l-tyrosine (NNPT), retrieved from the Protein Data Bank (PDB) (https://www.rcsb.org/) under the accession code 8I7P.^29^ This specific structure was selected because NNPT is, to date, the most efficient competitive inhibitor developed and tested *in vitro*, with a half-maximal inhibitory concentration (IC_50_) of 6 μM,^26,29^ making it a promising candidate as a warhead for designing a PROTAC to recruit RTA. It is worth noting that an IC_50_ in the μM range, although insufficient for effective inhibition, may still be adequate to enable PROTAC activity. Since PROTAC technology only requires ligands that can temporarily promote ternary complex formation, even low-affinity binders of the POI can be successfully incorporated into PROTACs.^30,31^

The experimental structures of E3 ligases used in this work are available in the PDB (https://www.rcsb.org/) under the codes 5NVV^7^ and 8OIZ^32^ and contain, respectively, Von Hippel–Hippel Lindau (VHL) complexed with inhibitor VHL3 and cereblon (CRBN) complexed with pomalidomide. Those structures have already been reported in PROTAC design studies in the literature.^7,32–34^

The protein structures were optimized using the *QuickPrep* tool from the MOE software package (https://www.chemcomp.com/Products.htm) by repairing gaps, adjusting bond lengths and angles, calculating charges, and appropriately protonating residues based on physiological pH. Crystallographic water molecules and artifacts were removed, resulting in an optimized form of each protein in complex with the respective inhibitors for use in the subsequent theoretical studies.

### PROTAC Design

The rationale for the design of the PROTACs used in this work is illustrated in Figure 2. The attachment points to VHL and CRBN ligands were the same connection sites previously reported in PROTAC design works.^33,34^ It is recognized that the conjugation point (exit vector) of the linker on the E3 ligase binders can significantly influence the resulting ternary complex. To maintain the scope of this computational Proof-of-Concept study and considering the exploration of five distinct linkers, we strategically opted to use the most well-established conjugation points found in the literature for the selected ligands. This selection is based on the dominance of these exit vectors in PROTAC libraries,^35^ ensuring that the proposed designs are built upon a validated foundation and allowing the computational effort to focus primarily on linker variation and the exit vector for the RTA ligand, as explained below.

**Figure 2:**
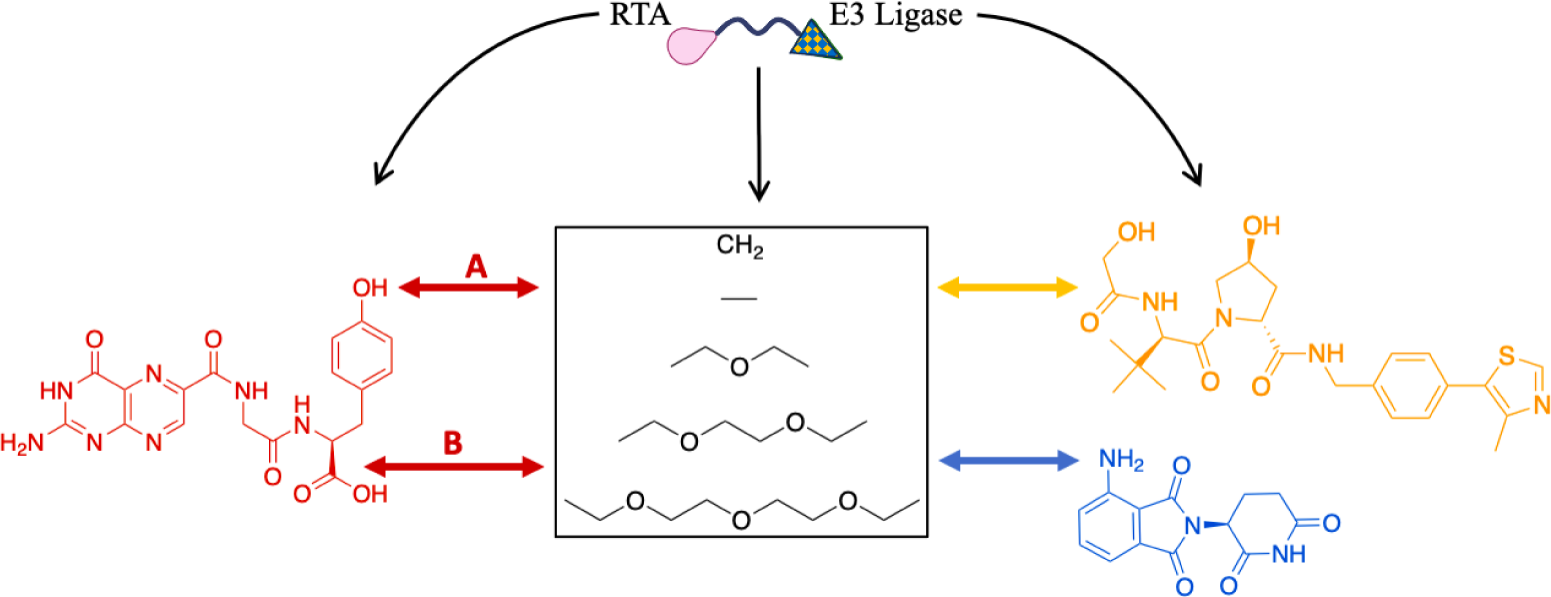
Design of PROTACs targeting RTA. The double arrows show the connection points between the warheads and linkers.

As for NNPT (RTA inhibitor), the connection site was determined by identifying its most solvent-exposed regions within the RTA active site (Figure 3A), and also considering synthetic feasibility to facilitate future experimental validation. Two potential attachment sites were identified: the oxygen atom of the phenol and the carbon of the carboxylic acid group, both highlighted with yellow circles in Figure 3B. The linkers were defined based on the most common and explored PROTAC linkers, which consist of alkyl or polyethylene glycol (PEG) units^8,34,36^ of variable lengths (see Figure 2). The structures of the PROTACs designed according to the criteria established above are illustrated in Figures 4 and 5.

**Figure 3:**
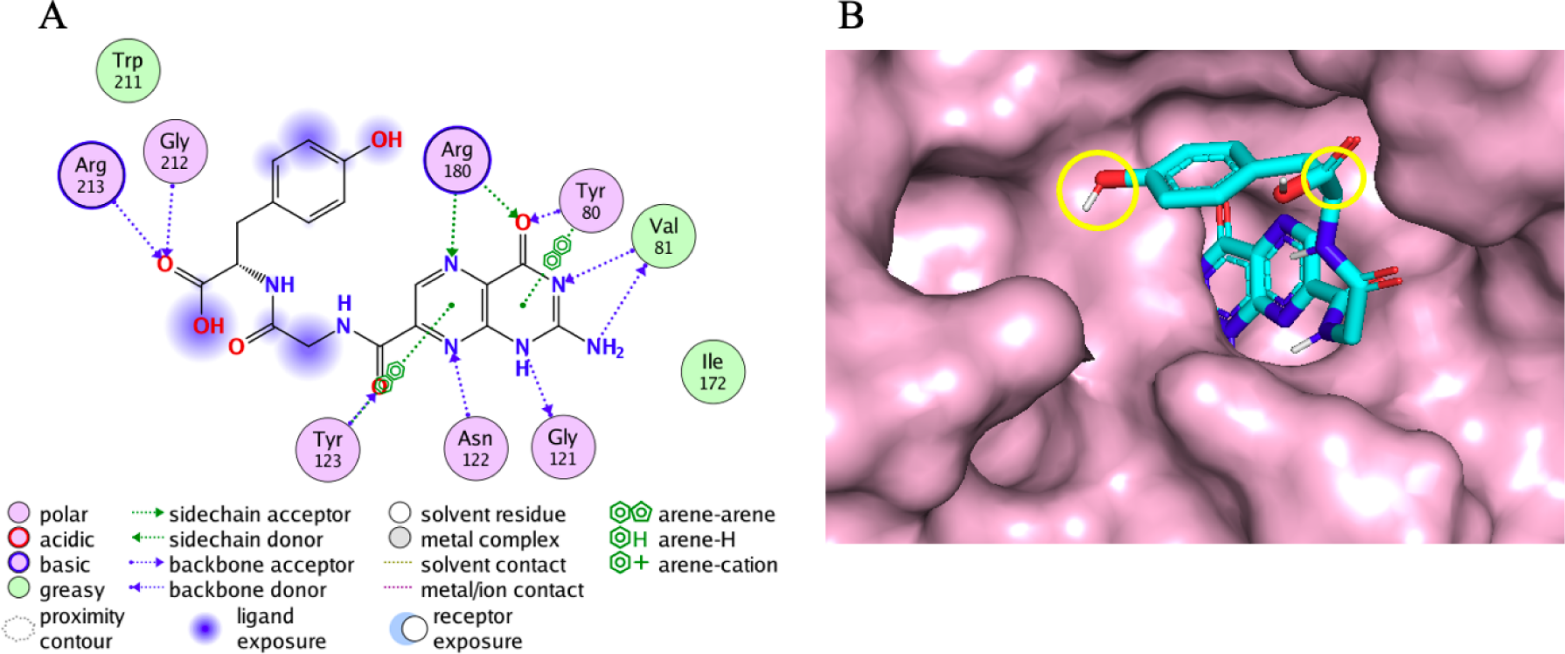
**A)** 2D map of the NNPT interactions inside RTA (from PDB ID: 8I7P^29^); solvent exposed parts are shown under blue shadows. **B)** NNPT inside the RTA (represented as a pink surface) active site; points of connection between NNPT and linkers are circled in yellow.

**Figure 4:**
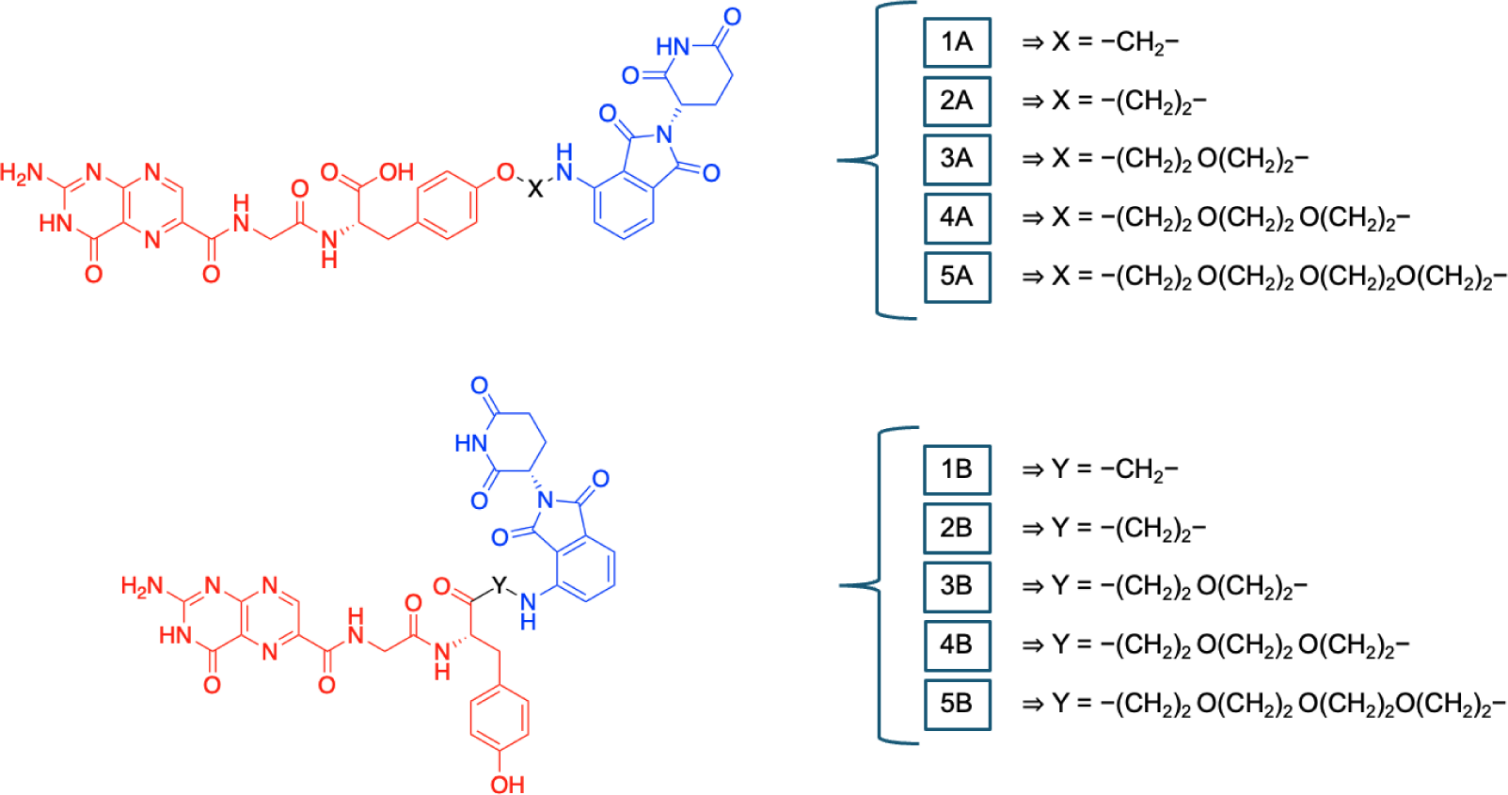
CRBN-recruiting PROTACs. The RTA recruiting part is shown in red, the linker in black, and the CRBN-recruiting part in blue.

**Figure 5:**
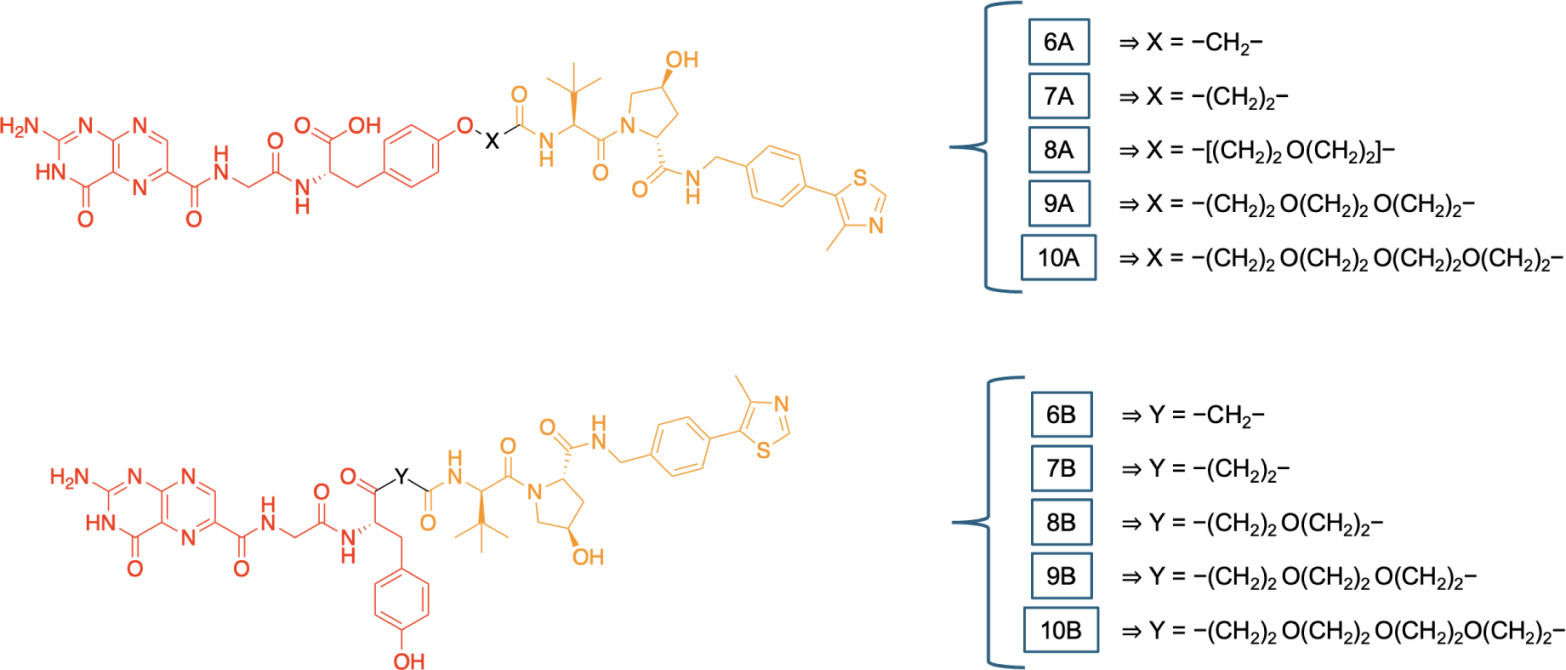
VHL-recruiting PROTACs. The RTA-recruiting part is shown in red, the linker is in black, and the VHL-recruiting part is in orange.

The 3D structures of the PROTACs were constructed and optimized up to a root-mean-square (RMSD) gradient of the potential energy below 0.1 kcal/mol Å^37^ using the “Builder” tool of MOE (https://www.chemcomp.com/Products.htm). Afterward, each PROTAC was exported to an individual database in the .mdb format to be used in the conformational search and prediction of ternary complexes.

### Assembly of the Ternary Complexes

For each tested PROTAC and E3 ligase, the ternary complexes (RTA-PROTAC-E3) were assembled using the Method 4B of the conformational searching and scoring protein-linker-protein tool^27,28^ implemented within MOE (https://www.chemcomp.com/Products.htm) and following the protocol described by Franca and coworkers.^12^ The generated ternary complexes were subjected to a restrained minimization protocol, in which the PROTAC and protein side chain atoms were unrestrained, while the protein backbone atoms were minimized as separate rigid bodies. After minimization, an all-against-all RMSD matrix based on protein carbons was generated for all final ternary complexes, and the structures were clustered by using a 10 Å cutoff. The most populous cluster was selected, and within it, the ternary complex with the lowest PROTAC internal energy was chosen as the representative structure for subsequent MD simulations since a less energetic conformation of the molecule is more feasible and therefore more likely to occur under experimental conditions.

### MD Simulations

MD simulations were prepared using MOE (https://www.chemcomp.com/Products.htm) to generate input files for NAMD2 ^38,39^ via the Compute → Simulations → Dynamics workflow. The AMBER19 force field^37^ was used for proteins, and the Extended Hückel Theory (EHT)^40^ was applied for the small molecules. A 10 Å cutoff was used for electrostatic interactions, and a switching distance of 8 to 10 Å was set for van der Waals interactions. Each ternary complex was solvated in a periodic water box containing nearly 16,000 water molecules for complexes with VHL and 27,000 water molecules for complexes with CRBN, neutralized with NaCl ions, and subjected to energy minimization followed by heating to 310 K, in phases of 50 ps each. The systems were equilibrated by using NVT and NPT ensembles for two phases of 200 ps each. The production steps were then run for 100 ns at 310 K and 1 atm. The resulting trajectories were analyzed using the MD Analysis tool in MOE (https://www.chemcomp.com/Products.htm) and Visual Molecular Dynamics (VMD).^41^

Additionally, the best performing complexes had their production times extended up to 200 ns, to ensure the stability and the overall behavior observed in the first 100 ns. All MD simulations were carried out in triplicates.

## Results and Discussion

### Clustering

Tables 1 and 2 summarize the total number of ternary complexes predicted for CRBN and VHL, respectively. Overall, the data indicate that larger PROTACs tend to form more ternary complexes, which is expected since increased linker length confers greater flexibility, allowing the molecule to adopt multiple conformations that stabilize the protein–protein assembly. This same flexibility, however, also leads to a higher diversity of conformations, reflected in the formation of multiple clusters. This trend is particularly evident for CRBN (Table 1): as linker size increases, the number of ternary complexes rises, but the percentage grouped in the most populated cluster decreases due to the emergence of additional clusters. In contrast, the VHL complexes did not follow this pattern. Notably, PROTACs 8A and 9B showed a high proportion of ternary complexes concentrated in the main cluster (Table 2), suggesting a greater likelihood that this assembly could occur experimentally.

**Table 1:** Docking Results of Ternary Complexes RTA-PROTAC-CRBN

PROTAC name	Number of ternary complexes formed (total population)	Number of different clusters formed	Number of ternary complexes in the largest cluster formed	% of population in largest cluster	PROTAC-proteins binding energy (kcal/mol) of the selected ternary complex
1A	0	0	0	—	—
2A	0	0	0	—	—
3A	2	2	1	50.0%	–16.1
4A	71	13	15	21.1%	–14.8
5A	313	48	44	14.1%	–18.4
1B	0	0	0	—	—
2B	9	2	5	55.6%	–15.8
3B	59	8	23	39.0%	–16.3
4B	298	34	47	15.8%	–16.9
5B	805	85	74	9.2%	–16.7

**Table 2:** Docking Results of Ternary Complexes RTA-PROTAC-VHL

PROTAC name	Number of ternary complexes formed (total population)	Number of different clusters formed	Number of ternary complexes in the largest cluster formed	% of population in largest cluster	PROTAC-proteins binding energy (kcal/mol) of the selected ternary complex
6A	1	1	1	100%	–16.0
7A	8	6	3	37.5%	–16.5
8A	73	9	34	46.6%	–21.1
9A	140	10	29	20.7%	–18.9
10A	884	42	192	21.7%	–20.1
6B	9	7	2	22.2%	–16.8
7B	8	6	2	25.0%	–18.1
8B	27	9	10	37.0%	–21.4
9B	94	17	40	42.6%	–20.0
10B	671	72	128	19.1%	–20.6

The last columns in Tables 1 and 2 report the binding energy between the PROTAC and both proteins within the representative ternary complex selected for MD simulations. This complex corresponds to the most populated cluster and features the PROTAC in its lowest-intermediate conformation, as described in the Assembly of the Ternary Complexes subsection of the Methods section. As can be seen, the complexes with VHL presented, in general, more negative interaction energies than complexes with CRBN, indicating a possible higher stability.

The shortest CRBN-recruiting PROTACs (1A, 2A, and 1B) were unable to form successful ternary complexes with CRBN and RTA. This probably occurred because CRBN is a much larger protein in comparison with RTA and VHL, so steric clashes and surface incompatibilities between RTA and CRBN become more important in a way that a larger PROTAC is needed to accommodate these two proteins in a favorable orientation. Figure 6 shows the selected complexes RTA-8A-VHL and RTA-3A-CRBN (which have the same linker length) to illustrate the size difference of the proteins and help us understand why only larger PROTACs seem to be able to bring RTA and CRBN together.

**Figure 6:**
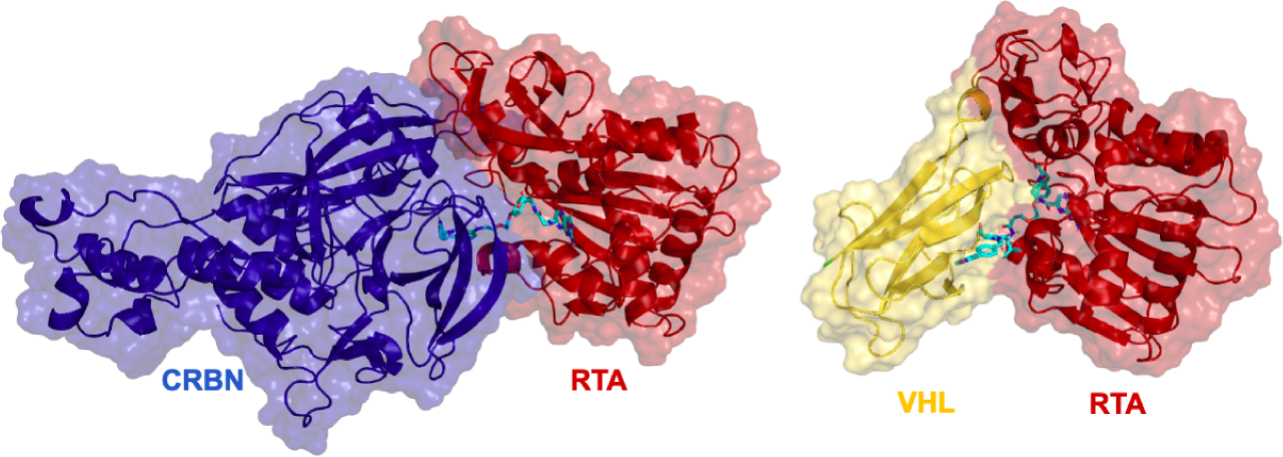
Ternary complexes of RTA-3A-CRBN (left) and RTA-8A-VHL (right) selected after running the conformational searching and scoring protein-linker-protein tool^27,28^ of MOE (https://www.chemcomp.com/Products.htm). Proteins are shown in cartoon inside their surfaces, while the PROTACs are shown in sticks.

To assess the geometric versatility and identify preferred binding modes of the E3 ligases when recruited to RTA, the representative ternary complexes derived from the most populated MD clusters were visually analyzed. The alignment, using RTA as the reference, allows direct comparison of the relative orientation of CRBN or VHL across all PROTACs tested.

Figures 7 and 8 depict the representative ternary complexes superposed. In each case, RTA, the PROTAC, and the corresponding E3 ligase (CRBN or VHL) are colored consistently to facilitate differentiation among complexes, and RTA is used as a reference for superposition to facilitate the comparison. For CRBN-containing complexes, no predominant binding mode was observed, as CRBN adopts different orientations relative to RTA (Figure 7). Nevertheless, certain PROTACs promoted similar arrangements, such as 3A and 5A (Figure 7.I and III), which yielded comparable binding modes of the proteins. In contrast, group B PROTACs (2B, 3B, 4B, and 5B) each adopted distinct conformations, leading to unique positions of CRBN with respect to RTA in their respective ternary complexes.

**Figure 7:**
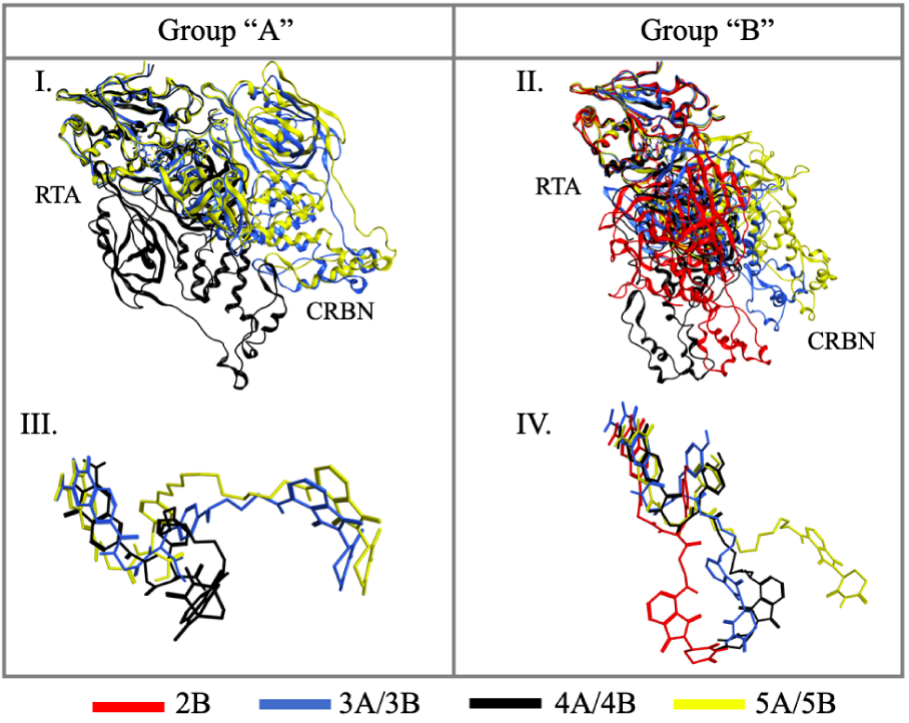
Overlap of the best ternary complexes selected from the most populated clusters involving CRBN. I and II: overlap of the whole ternary systems; III and IV: overlap of the PROTACs without the proteins. Proteins are represented as ribbons and PROTACs in stick.

**Figure 8:**
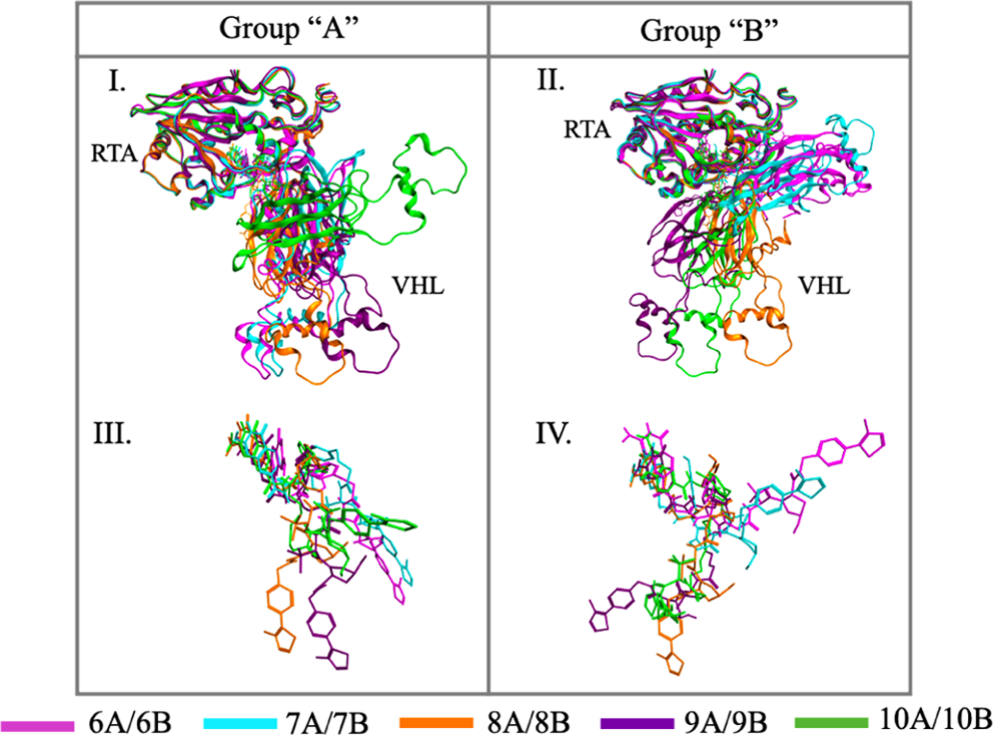
Overlap of the best ternary complexes selected from the most populated clusters involving VHL. I and II: overlap of the whole ternary systems; III and IV: overlap of the PROTACs without the proteins. Proteins are represented as ribbons and PROTACs in stick.

As for complexes involving VHL (Figure 8), clearer patterns can be observed. Among group A, only 10A, the longest and most flexible member of this family, induced a distinct ternary complex. The other four PROTACs adopted conformations that led to a similar relative arrangement of RTA and VHL, suggesting that this may represent a preferred mode of binding between the two proteins. Within group B, two distinct patterns emerged: the smallest PROTACs, 6B and 7B, formed similar ternary complexes, whereas the larger ones (8B, 9B, and 10B) clustered into another group, yielding complexes that were comparable to each other but clearly distinct from those involving 6B and 7B.

Additionally, in order to quantify the difference among the E3 ligase conformations shown in Figures 7 and 8, Figure 9 shows the pairwise α-carbon RMSD values for each E3 ligase in the different adopted conformations and orientations in all ternary complexes.

**Figure 9:**
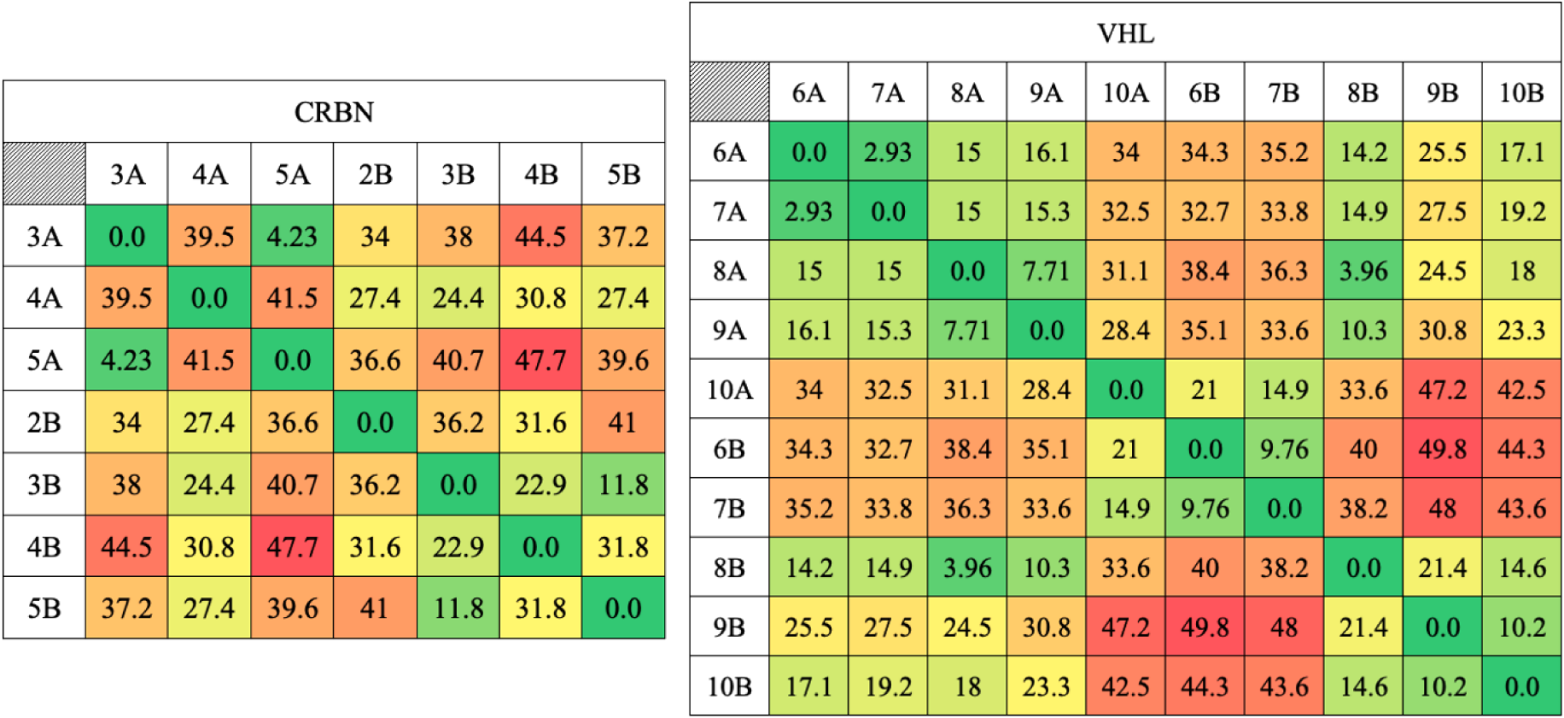
Pairwise α-carbon RMSD values for each E3 ligase in the representative ternary complex for each PROTAC. Lowest values are shown in green, and highest values in red.

Regarding CRBN, Figures 7 and 9 demonstrate that this E3 ligase exhibits high orientational versatility when recruited to RTA. Specifically, only PROTACs 3A and 5A induce a visually and quantitatively similar preferred orientation of CRBN relative to that of RTA within the ternary complex. Conversely, all other tested PROTACs appear to promote a unique, distinct preferred orientation for CRBN recruitment, highlighting the sensitivity of the CRBN/RTA interface to even minor changes in the PROTAC linker geometry.

The comparative structural analysis, encompassing Figures 8.I, II and 9, allows us to infer the existence of three predominant orientations of VHL relative to RTA within the ternary complex. Initially, visual inspection of Figure 8.I and II suggests that PROTACs 6B, 7B, and 10A result in the same VHL orientation. However, quantitative analysis of the carbon α RMSD values (Figure 9) is crucial, as it clearly elucidates that these arrangements are not identical, effectively delineating three distinct geometric patterns. The first is a primary arrangement, promoted by the majority of the molecules (PROTACs 6A, 7A, 8A, 9A, 8B, 9B, and 10B). The second is a specific secondary orientation, stabilized by PROTACs 6B and 7B. And the third is a unique orientation, promoted exclusively by PROTAC 10A. This distinction, resolved through RMSD analysis, underscores that the versatility of the linker enables the stabilization of distinct arrangements which, without quantitative validation, might otherwise appear geometrically similar upon initial visual inspection. These observations suggest that VHL recruitment by RTA is not random but tends to stabilize into a limited set of geometries, with one orientation being favored by most of the PROTACs.

### MD Simulations

After MD simulations, the complexes were analyzed in terms of RMSD, per-residue protein fluctuations (root-mean-square fluctuations—RMSF), PROTAC-Protein binding energies, and hydrogen bonding patterns.

Figure 10 shows binding energy values between each PROTAC and the proteins, and the RMSD values of RTA, CRBN, and PROTAC for all complexes involving CRBN. No results are reported for PROTACs 1A, 2A, and 1B, as these molecules failed to form stable complexes with RTA and CRBN. All results are presented as mean values (lines), with shaded areas in the same color indicating the standard error of the mean (SEM) across the MD triplicates.

**Figure 10:**
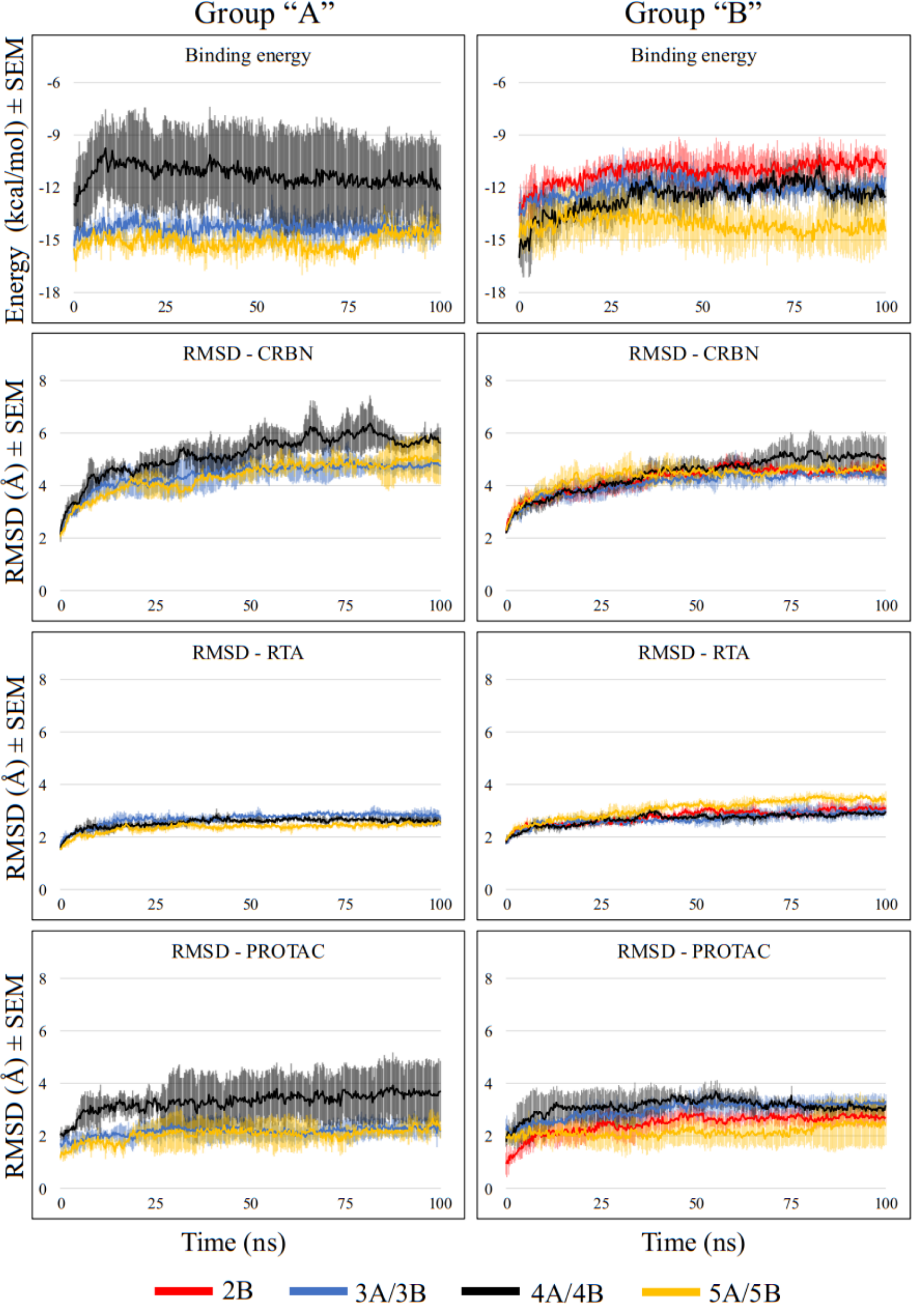
MD results of PROTACs recruiting CRBN. Each line represents the mean value between the three MD replicates, and the lighter color above and below the line represents the standard error of the mean (SEM).

Binding energy values (Figure 10, first row of charts) highlight 3A and 5A as the ligands with the most negative energies, showing high consistency across the three MD replicates. These results indicate more favorable and potentially more realistic interactions for these PROTACs. Notably, 3A, 5A, and also 5B were the only PROTACs that sustained binding energy values as negative as, or at times even more negative than, those predicted in the ternary complex docking simulations (Table 1).

The RMSD plots show that in all systems, protein and ligands achieved stability after nearly 12 ns of simulation, where a horizontal line with no fluctuations over 2 Å between frames is observed. The RMSD values for CRBN and RTA were highly consistent across all MD runs regardless of the PROTAC bound. This behavior is expected, as the proteins are much larger and structurally more rigid than the PROTACs, resulting in lower flexibility. The overall stability observed for both proteins supports the likelihood that these assemblies could form experimentally. Nevertheless, some deviations were noted, particularly for CRBN when complexed with PROTACs 4A and 5A, and for RTA with PROTAC 5B, which exhibited higher RMSD values. These fluctuations suggest reduced stability of the corresponding ternary complexes, indicating that such assemblies may be less favorable in experimental conditions.

Figure 10 indicates that 2B, 3A, and 3B achieved greater stability, maintaining steadier positions throughout the simulations. This behavior can be partially attributed to their shorter size, which confers fewer degrees of freedom and lower flexibility. However, PROTACs 4B and particularly 4A exhibited the largest positional fluctuations, despite being shorter and theoretically less flexible than 5A and 5B, which performed better but showed more fluctuations compared to 2B, 3A, and 3B. This suggests that PROTAC size alone does not necessarily predict stability within the ternary complex.

In general, PROTAC 4A presented the poorest MD results, displaying less negative binding energy values, higher positional fluctuations, and lower consistency among MD triplicates, as indicated by the gray shadows around the black lines in Figure 10. In contrast, PROTAC 3A emerged as the most promising candidate, yielding the best MD outcomes.

It is interesting to note that the results from the Ternary Complex Docking (Method 4B) and MD simulations provide complementary rather than contradictory, insights. Method 4B, which evaluates conformational sampling and pose diversity, suggested that PROTAC 3A is relatively ineffective, yielding only two favorable ternary complex models. This low sampling capacity is attributed to its smaller size and lower intrinsic flexibility compared to larger PROTACs, thereby limiting the number of stable arrangements that it can mediate. In contrast, MD demonstrated that the preferred binding mode of PROTAC 3A is highly stable over 100 ns, showing a favorable positional stability profile represented by RMSD values. This observation suggests that although PROTAC 3A exhibits low conformational versatility in complex formation, the specific arrangement it achieves is thermodynamically robust enough to be maintained over time, preserving it as a promising degrader candidate.

Figure 11 illustrates the hydrogen bonds (H-bonds) formed between these two PROTACs and the proteins during the MD simulations expressed as occupancy percentages. PROTAC 4A established far fewer H-bonds, particularly with CRBN (blue columns), which is consistent with its poorer stabilization within the ternary complex. PROTAC 3A, on the other hand, formed multiple H-bonds with higher occupancies across various residues of both proteins, further suggesting that it may represent a promising candidate for future experimental validation.

**Figure 11:**
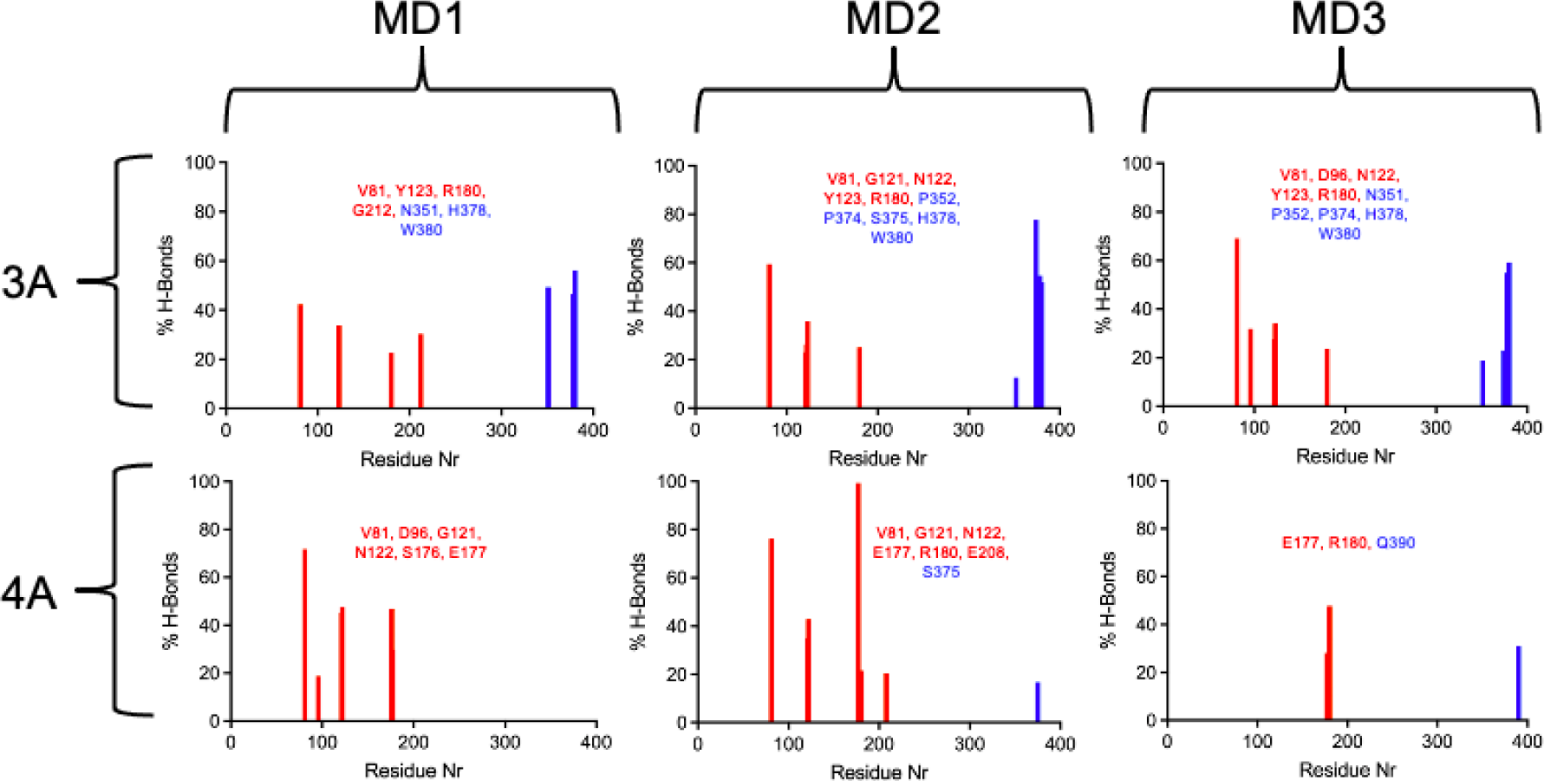
% of H-bonds per residue formed between 3A and 4A and the proteins during the MD simulations. Red bars correspond to RTA residues, and blue ones correspond to CRBN residues. Only H-bonds that are prevalent for more than 10% of the simulated time are shown.

The H-bond occupancies of the other PROTACs are presented in Figure S1. In general, group “B” ligands performed worse than group “A” in terms of H-bond formation, and 4B presented poorer results in this matter. It is also worth noting that 4A and 4B, both bearing a two-unit PEG linker, generally performed worse than the other CRBN-recruiting PROTACs, suggesting that this linker length may be less favorable in this context.

Figure 12 presents the MD results for ternary complexes RTA-PROTAC-VHL over the MD simulations. The variation in binding energy, shown in the first row of charts, indicates that PROTACs bearing PEG linkers (8A, 8B, 9A, 9B, and 10B) generally exhibited more negative energy values than those with alkyl linkers. The exception was PROTAC 10A, which showed less negative binding energy values throughout the MD simulations. This suggests that although PEG linkers can promote additional hydrogen bonding due to their oxygen atoms, this feature alone is not sufficient to ensure greater stability of the PROTAC within the ternary complex.

**Figure 12:**
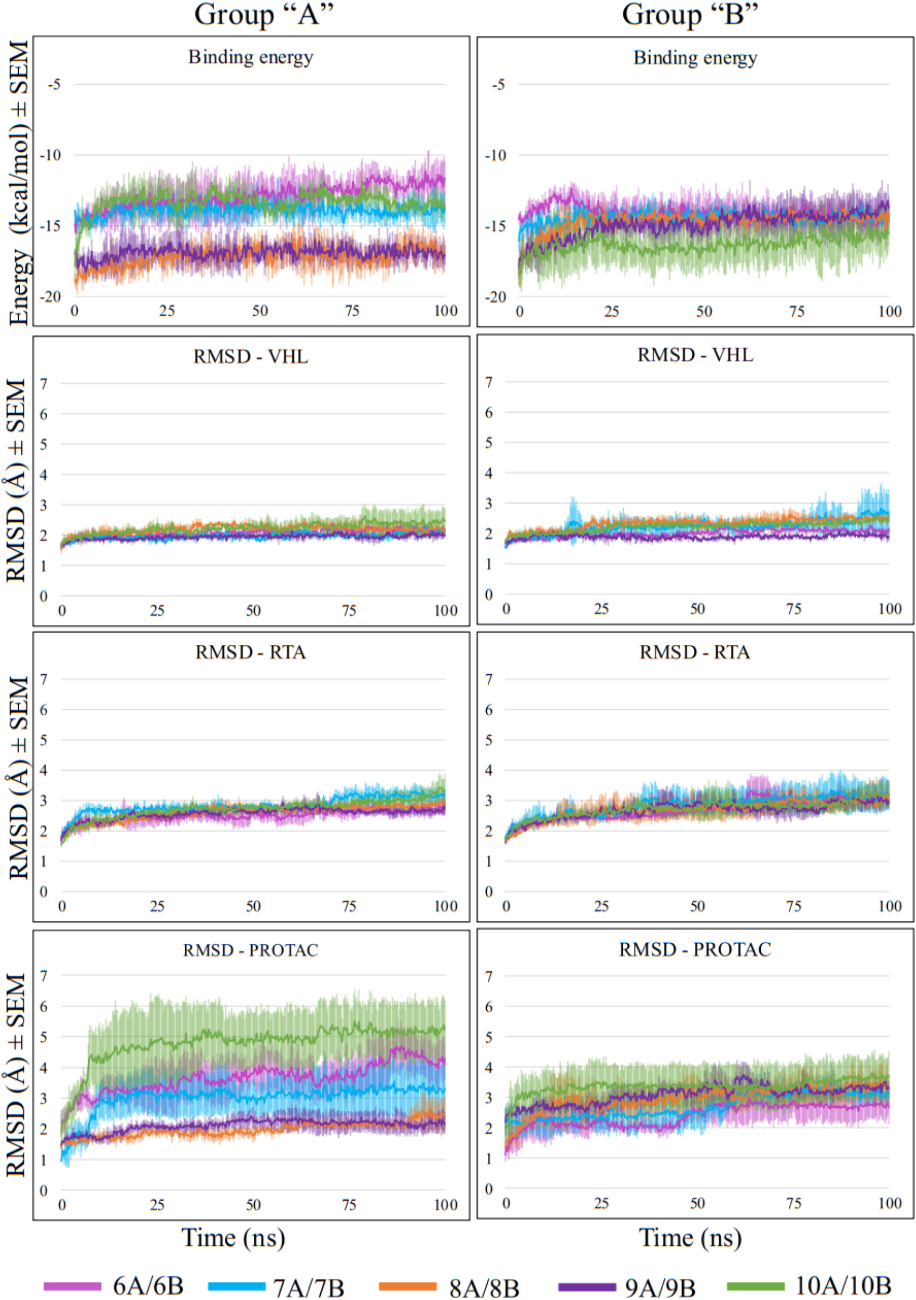
MD results of PROTACs recruiting VHL.

RMSD values for VHL and RTA (Figure 12, second and third rows) were highly consistent across all systems, as previously observed for CRBN. All protein RMSD values generally fluctuated below 3 Å with mostly horizontal profiles, indicating good stability within the complexes. Notably, the data also suggest that PROTACs 8A and 9A have higher capacities to maintain RTA stability in the ternary assemblies. Analysis of their RMSD profiles (last row of charts of Figure 12) stands out as the most stable, displaying minimal positional fluctuations. Interestingly, 10A, which has a similar but longer PEG linker, presented a worse performance, indicating that there might be an optimal linker length of one or two PEG units for PROTACs recruiting VHL and RTA. PROTAC 10B, possessing the same linker length as 10A, exhibited superior mean binding energy values; however, the considerable SEM ranges observed indicate notable variability among the MD triplicates, suggesting reduced reproducibility and a less predictable dynamic behavior for this PROTAC, which may be attributed to its higher degree of flexibility due to a longer linker. Finally, PROTAC 6A, the shortest PROTAC, performed poorly, displaying unstable RMSD profiles and less negative interaction energies, indicating that such a short linker may not be interesting either and that an optimum linker length exists, as expected.^34,36^

It is notable that certain PROTACs, particularly 4A, 10A, and 10B, exhibit large SEM envelopes (Figures 10 and 12). This significant variability across replicates highlights the stochastic nature of MD simulations and the presence of multiple accessible binding modes in the conformational landscape. A large SEM indicates that the averaging of trajectories reflects a highly heterogeneous ensemble of stable or semistable conformations, suggesting that the PROTAC possesses high conformational versatility capable of bridging the RTA/E3 interface via distinct structural arrangements. While this reduces the predictive power for a single, unique binding mode, it confirms the PROTAC’s ability to maintain the stability of the ternary complex through dynamic structural exploration.

Figure 13 presents the H-bond occupancies of 6A (the ligand with the worst MD performance) compared to 8A and 9A (the ligands with the best MD performances). Unlike what was observed in the RTA-PROTACs-CRBN complexes (Figure 11), the worst-performing ligand (6A) here was able to form a reasonable number of H-bonds with both proteins. However, these interactions were insufficient to maintain the stability of the ternary complex.

**Figure 13:**
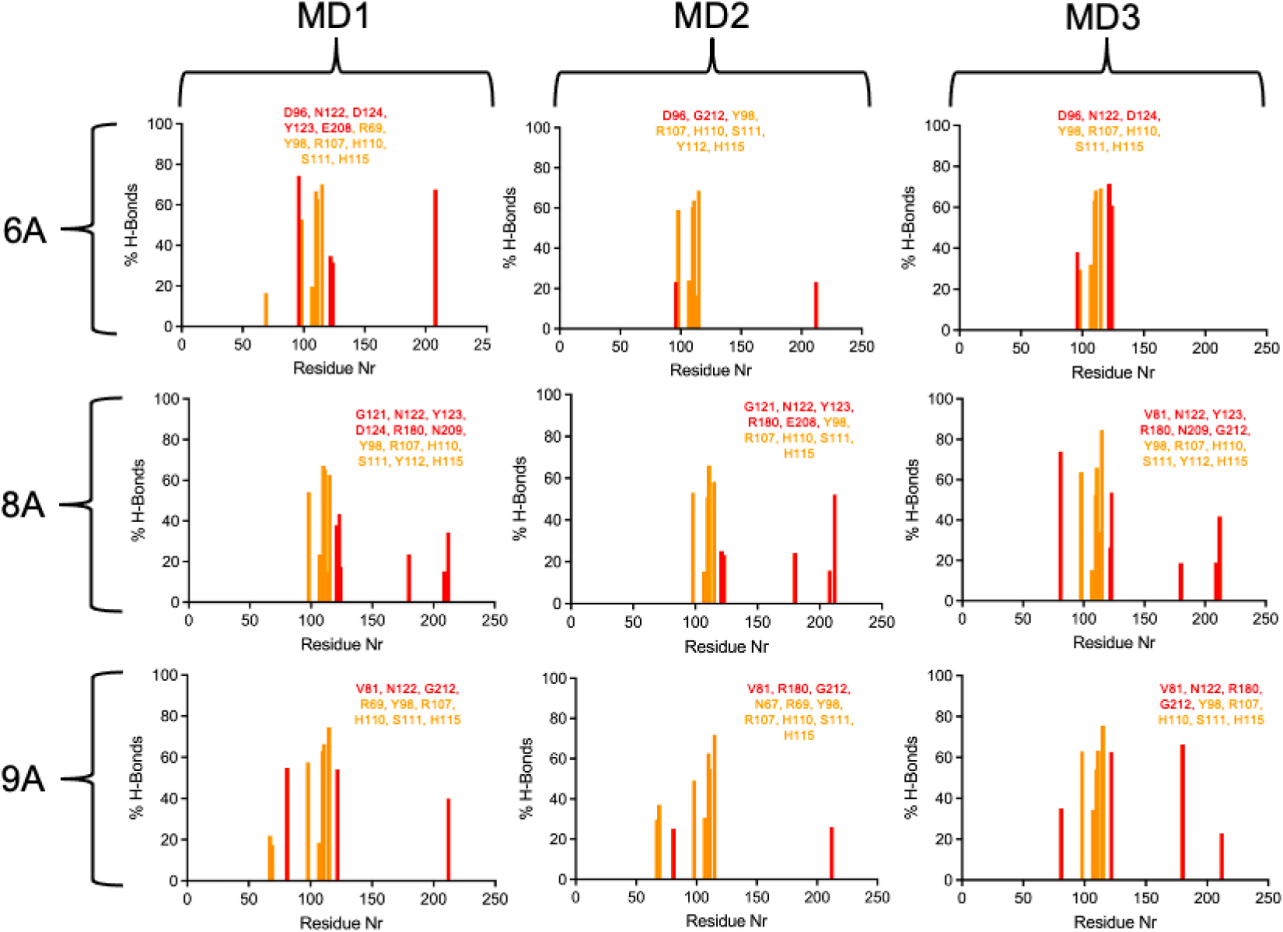
% of H-bonds per residue formed between 6A, 8A, and 9A and the proteins during the MD simulations. Red bars correspond to RTA residues, and orange ones correspond to VHL residues. Only H-bonds prevalent for more than 10% of the simulated time are shown.

Figures S2 and S3 illustrate all PROTAC-RTA and PROTAC-VHL H-bonds formed during the MD simulations, in terms of their respective occupancy percentages. In general, all PROTACs (families “A” and “B”) established H-bonds with various protein residues and could maintain these interactions, which can be observed by the high occupancy values.

Figures S4 and S5 show data of RMSF per residue of CRBN and VHL, respectively, and RTA, while Figures S6 and S7 depict the positional variations of both proteins and PROTACs for all ligands submitted to 100 ns MD simulations. The “sausage” representation highlights protein fluctuations along the trajectory, with tube thickness proportional to residue RMSF values. The PROTACs are shown as superpositions of conformations extracted from different frames at each 1 ns of MD production, illustrating their mobility within the binding site. As expected, structurally rigid protein regions (helices and β-sheets) appear as thinner tubes.

Although CRBN displayed higher RMSF values than VHL (Figures 10 and 12), Figures S6 and S7 reveal that its most flexible region lies far from the active site and therefore distant from the PROTAC, suggesting that the ternary complex is not the main cause of this relative instability. It is also noteworthy that CRBN is substantially larger than VHL, which inherently contributes to higher RMSF values.

It is noteworthy that the simulation setup models only the binding domains of CRBN and VHL rather than their full E3 ligase complexes (CRL4^CRBN^ and CRL2^VHL^, respectively), which represents a limitation inherent to the computational complexity of modeling such large assemblies. The exclusion of key physiological partners, such as DDB1 for CRBN and Cullin 2/Elongin B/C for VHL, results in a nonphysiological environment for the E3 ligases. This setup likely leads to an overestimation of the flexibility in the interface subdomains of both CRBN (as evidenced by motion in the DDB1-binding region) and VHL.

While the inclusion of the full E3 complexes would likely reduce the absolute RMSF values by providing a stabilizing scaffold, the current setup remains appropriate for assessing the relative impact of the PROTACs. Crucially, because this intrinsic methodological flexibility is uniform across all CRBN systems and all VHL systems studied, the observed differential effects on E3 ligase stability induced by the various linkers remain valid indicators of their capacity to stabilize the target/E3 ligase interface in the ternary complex.

Regarding the PROTACs, the superposition of frames highlights notable differences in positional stability already observed in Figures 10 and 12. For instance, 4A and 10A exhibited greater conformational variability, resulting in poorer frame overlap, whereas 3A and 8A showed much tighter superposition, consistent with better positional stability.

MD results highlighted PROTACs 3A, 8A, and 9A as the most promising candidates, and their simulations were therefore extended to 200 ns to assess their long-term behavior. As shown in Figure 14, the favorable stability observed during the first 100 ns was maintained over the subsequent 100 ns, reinforcing the promising possibility of these molecules to show good experimental results.

**Figure 14:**
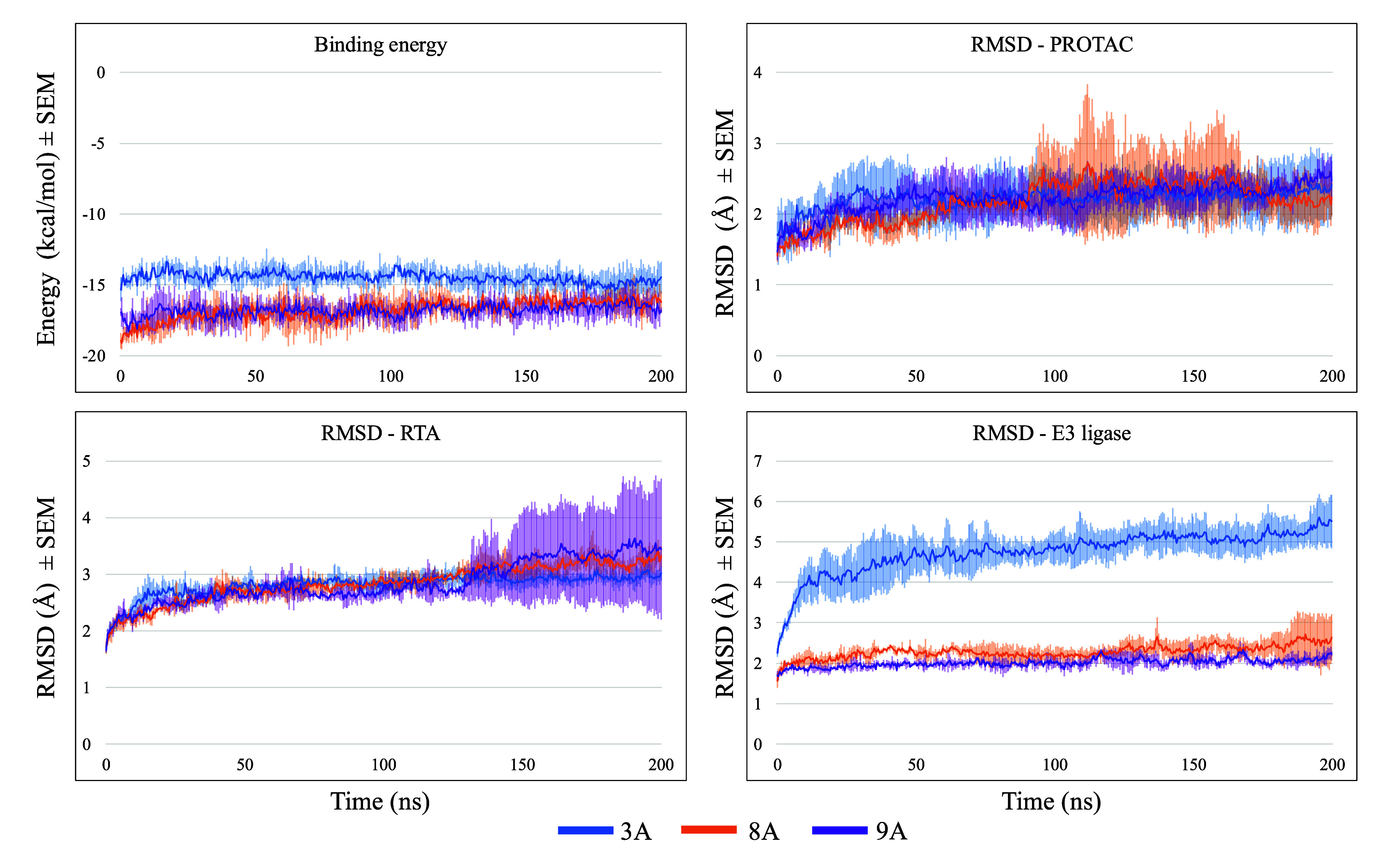
MD results of PROTACs submitted to 200 ns of simulation.

As previously observed, the protein behavior remained largely consistent across different PROTACs. The only exception was a single replicate of PROTAC 9A (purple line in Figure 14; RTA RMSD chart), where RTA exhibited greater positional variation that was reflected in the light purple shadow that represents the SEM. Since this deviation was not reproduced in the other two simulations, it is likely to represent a low-probability event.

Figure 15 illustrates the total number of H-bonds formed between every two species of the ternary complexes during the 200 ns of the MD simulation. As can be seen the number of H-bonds remained reasonably stable, confirming the stability of the ternary complexes. Regarding RTA-PROTAC (first column of charts in Figure 15), 8A and 9A showed a higher number of H-bonds compared to 3A. Since the RTA-binding moiety is the same for all three PROTACs (Figures 2 and 3), a higher number of H-bonds likely reflects a greater ability of the molecule to fit into RTA’s active site. 8A and 9A also outperformed 3A with VHL (second column in Figure 15); however, it is important to note that in this case both the E3 ligases and the PROTAC moieties engaging them are inherently different (Figures 4 and 5). Still, the three PROTACs maintained around three or more H-bonds with their corresponding E3 ligases for most of the simulation time over the three replicates, underscoring consistent interactions. Regarding the protein–protein H-bonds (third column), although the E3 ligases differ across cases, the results are noteworthy: multiple intermolecular H-bonds were sustained for almost the entire simulation time, indicating that RTA and the E3 ligases remained in close proximity, with amino acid residues frequently within ∼3 Å of each other, thereby enabling hydrogen-bond formation. Altogether, the H-bond profiles reinforce the stability of the ternary complexes in terms of intermolecular interactions. On average, 8A and 9A outperformed 3A, suggesting that VHL may be a more readily recruited E3 ligase than CRBN in this context.

**Figure 15:**
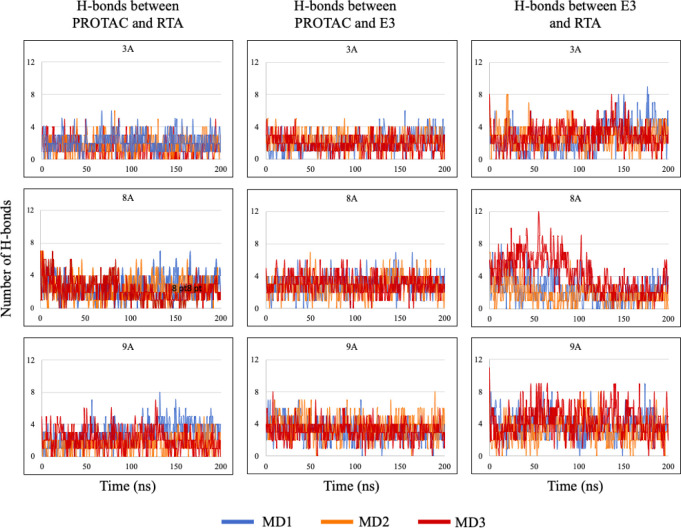
H-bonds between PROTACs and proteins during 200 ns MD simulations. The three lines in each graph represent the three replicates of the MD simulations.

Finally, Figure 16 depicts “sausage” representations of PROTAC and the proteins in the ternary complexes submitted to 200 ns MD simulations. The PROTACs are shown as superpositions of conformations extracted from different frames at every 2 ns of MD production, illustrating their mobility within the binding site. As expected, structurally rigid protein regions (helices and β-sheets) appear as thinner tubes.

**Figure 16:**
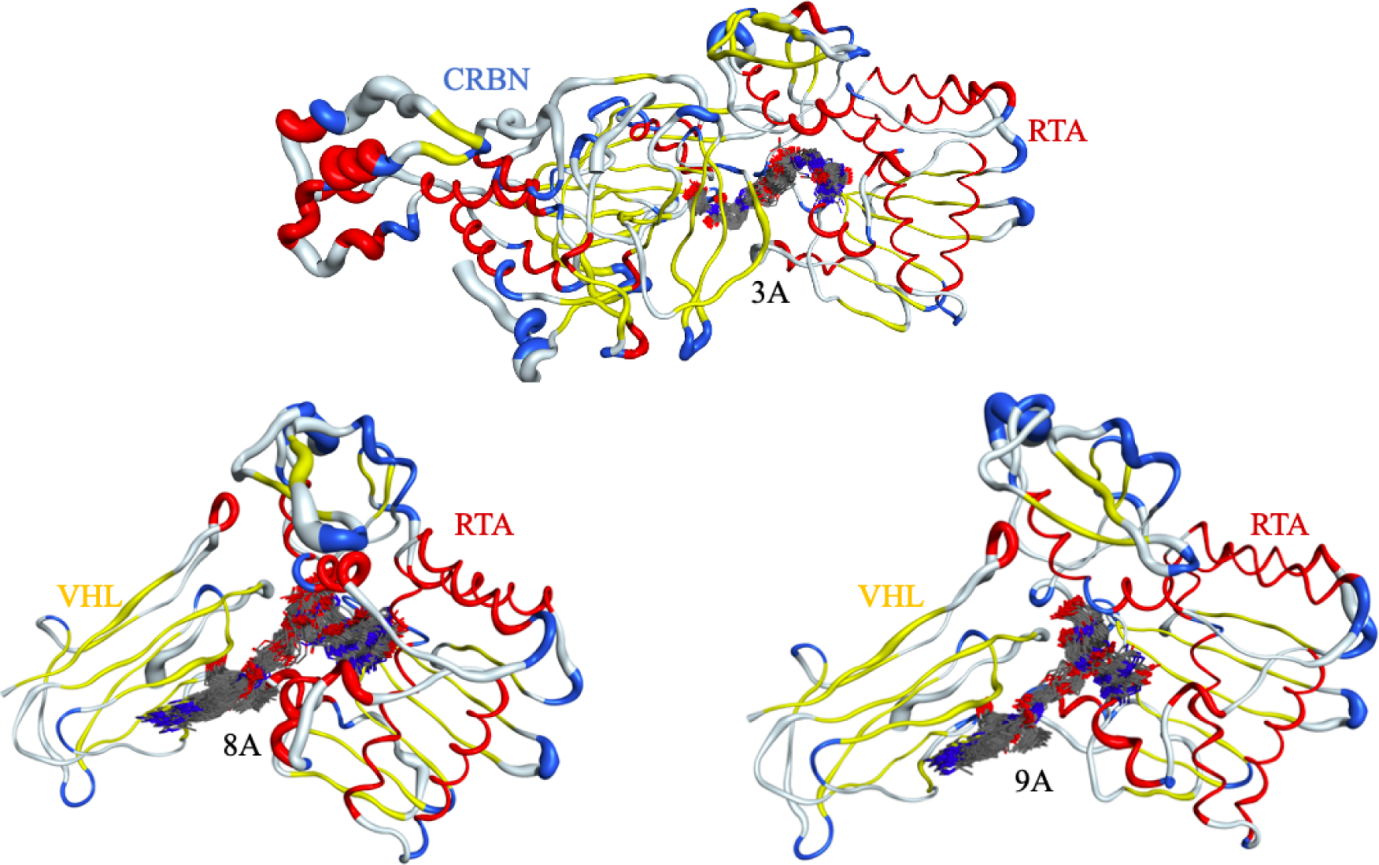
Sausage representation of protein fluctuations along the MD simulation, where the tube thickness is proportional to residue RMSF values. Secondary structure elements are colored as follows: α-helices in red, β-sheets in yellow, turns in blue, and loops in light gray. The PROTAC is displayed as a superposition of conformations extracted from different frames at 2 ns intervals of trajectory, illustrating its mobility within the binding site.

The behavior of the proteins was similar to the ones observed in Figures S6 and S7 for the 100 ns MD simulations. Regarding the PROTACs, the three ligands exhibited good stability and only minor positional variations, as indicated by the consistent superposition of the frames. PROTAC 8A showed slightly poorer performance compared to the other two, as revealed by both its frame superposition in Figure 16 and its RMSD profile in Figure 14.

## Conclusions

To the best of our knowledge, this work represents the first investigation of the PROTAC strategy toward the degradation of ricin. The computational results presented here still lack *in vitro* validation; however, they provide encouraging evidence that this approach could be feasible for neutralizing such a potent toxin. Our simulations identified PROTAC 3A (CRBN-recruiting) and PROTACs 8A and 9A (VHL-recruiting) as the most promising candidates, given their consistent stability, favorable interaction energies, and capacity to maintain the ternary complex integrity.

Among the recruited ligases, VHL appears more advantageous than CRBN, not only because two of the best-performing PROTACs (8A and 9A) were VHL-recruiting (compared to only one CRBN-based PROTAC, 3A) but also due to significant mechanistic differences observed in complex formation. Specifically, VHL complexes exhibited greater structural convergence and less positional dispersion (lower RMSD and higher pattern consistency in Figure 8) compared with CRBN complexes, which showed high orientational variability and greater conformational dispersion (Figures 7 and 9). This suggests that VHL promotes a more constrained and rigid binding mode when recruited to RTA, likely leading to more predictable and robust degradation *in vivo*.

Furthermore, in this context of RTA-targeting PROTACs, PEG linkers outperformed alkyl chains, with 1 or 2 PEG units emerging as the optimal configuration, since PROTACs with three PEG units exhibited reduced performance, which is also a desirable result due to the fact that larger molecules have less favorable drug-like properties.^42,43^

While further experimental validation is required, these findings establish a conceptual framework for expanding the use of targeted protein degradation beyond classical therapeutic targets, extending it to protein toxins of biological and defense relevance. This study therefore lays the groundwork for future efforts toward the rational design and optimization of PROTACs as a novel class of countermeasures against intoxication with ricin and related toxins.

## Supplementary Material





## Notes

Additional figures of computational results (PDF)
Input files of MD simulations and videos of MD trajectories of each system (ZIP)
