# Supplementary material for: PROTAC-Mediated Ternary Complex Stability with Ricin Toxin A: A Computational Perspective
Source: ACS Omega. 2026 Feb 9;11(7):11685–99. doi: 10.1021/acsomega.5c10223 (PMC12946957; doi:10.1021/acsomega.5c10223)
Supplement: Supplementary file 1 [file ao5c10223_si_001.pdf]

PROTAC-Mediated Ternary Complex Stability with Ricin Toxin A: A Computational Perspective

Fernanda D. Botelho[†], *Salim T. Islam*[‡,§], *Steven R. LaPlante*[‡,§], *Tanos C. C. Franca*[†,‡,§,∥,*]

[†]Laboratory of Molecular Modeling Applied to the Chemical and Biological Defense (LMCBD), Military Institute of Engineering, Rio de Janeiro, Rio de Janeiro 22290-270, Brazil

[‡]Institut National de la Recherche Scientifique (INRS), Centre Armand-Frappier Santé Biotechnologie, Université du Québec, Institut Pasteur International Network, Laval, QC H7V 1B7, Canada

[§]PROTEO, the Quebec Network for Research on Protein Function, Engineering, and Applications, Universite Laval, Quebec, QC G1V 0A6, Canada

[∥]Center for Basic and Applied Research, Faculty of Informatics and Management, University of Hradec Kralove, Rokitanskeho 62, Hradec Kralové 50003, Czech Republic

*Corresponding author: tanos@ime.eb.br

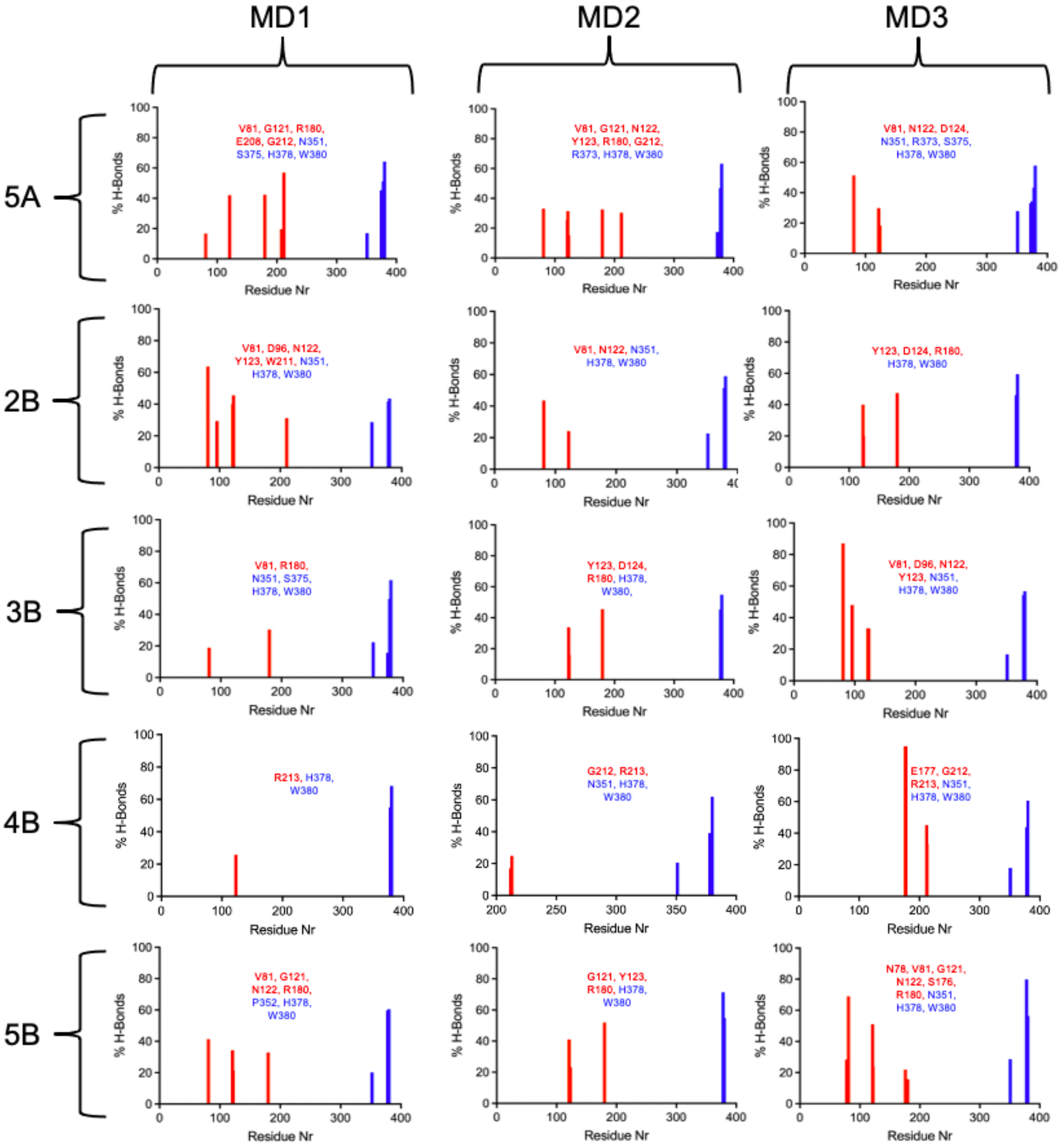

Figure S1. % of H-bonds per residue formed between PROTACs 5A, 2B, 3B, 4B, 5B and the target proteins during 100 ns of MD simulations. Red bars correspond to RTA residues, and blue ones correspond to CRBN residues. Only H-bonds prevalent for more than 10% of the simulated time are shown.

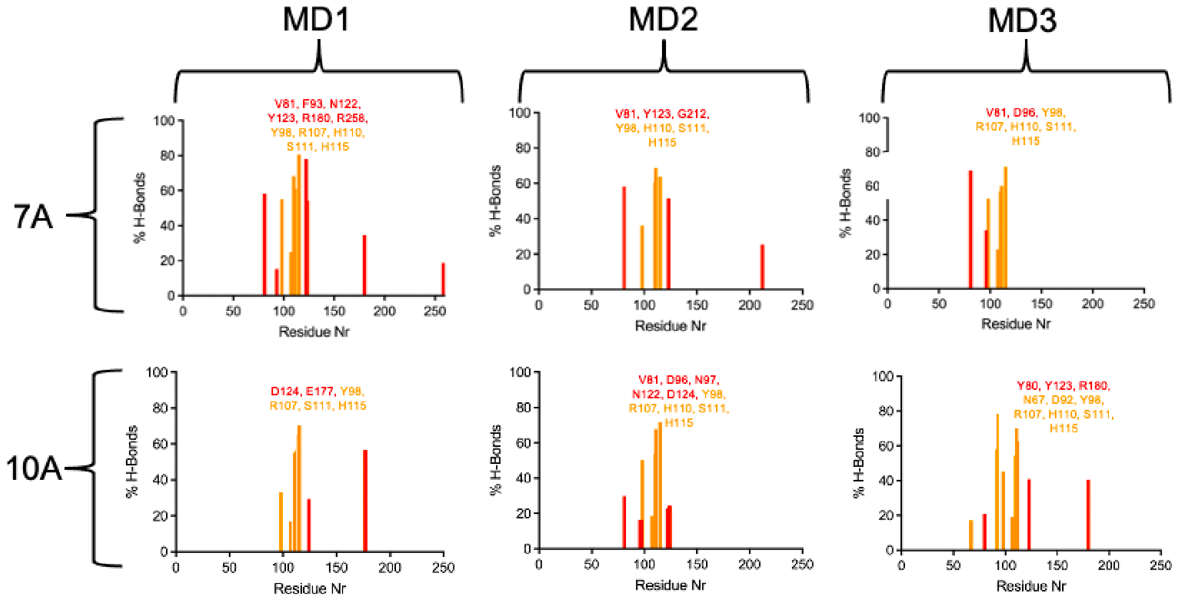

Figure S2. % of H-bonds per residue formed between PROTACs 7A, 10A and the target proteins during 100 ns of MD simulations. Red bars correspond to RTA residues, and orange ones correspond to VHL residues. Only H-bonds prevalent for more than 10% of the simulated time are shown.

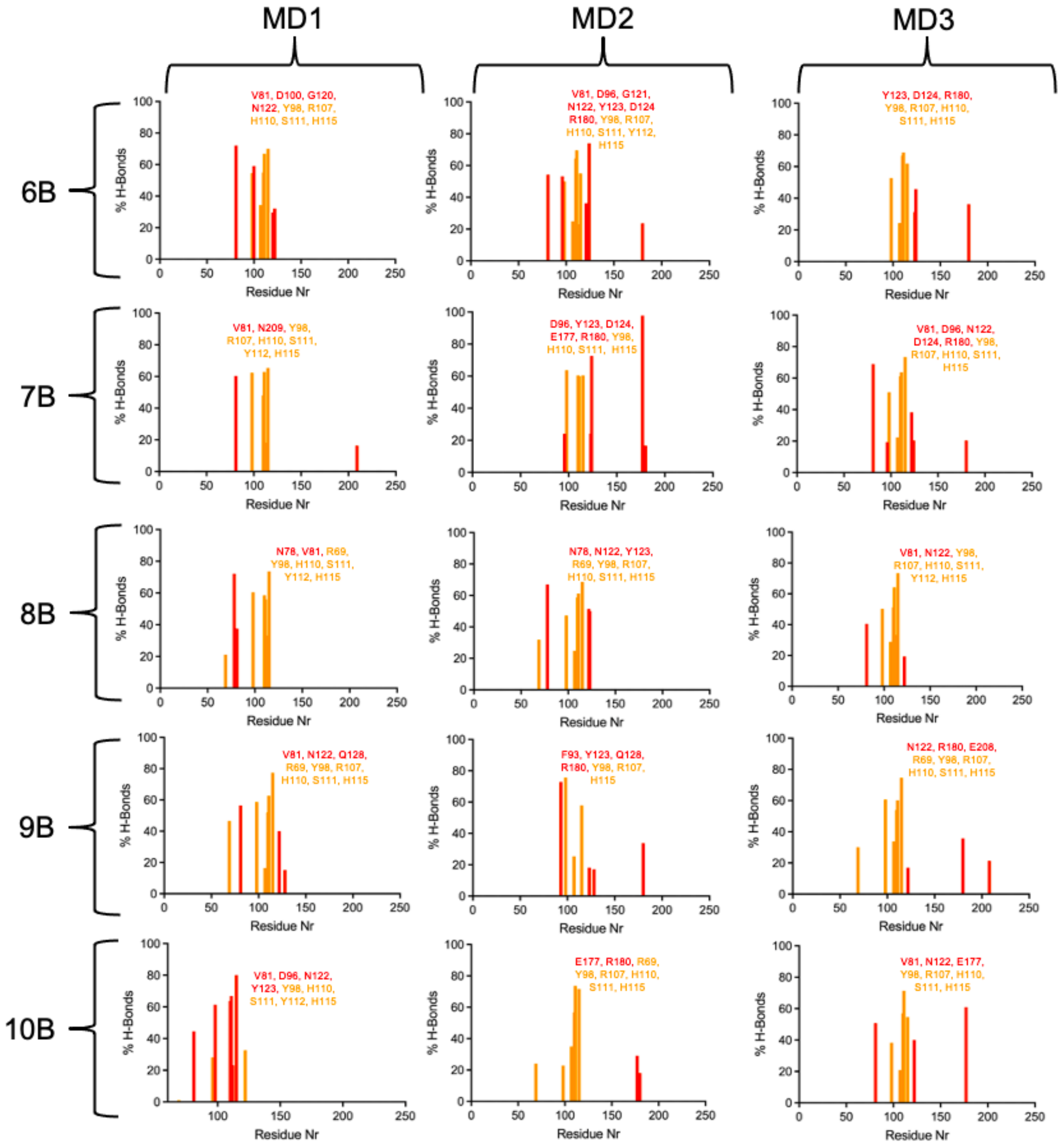

Figure S3. % of H-bonds per residue formed between PROTACs 6B – 10B and the target proteins during 100 ns of MD simulations. Red bars correspond to RTA residues, and orange ones correspond to VHL residues. Only H-bonds prevalent for more than 10% of the simulated time are shown.

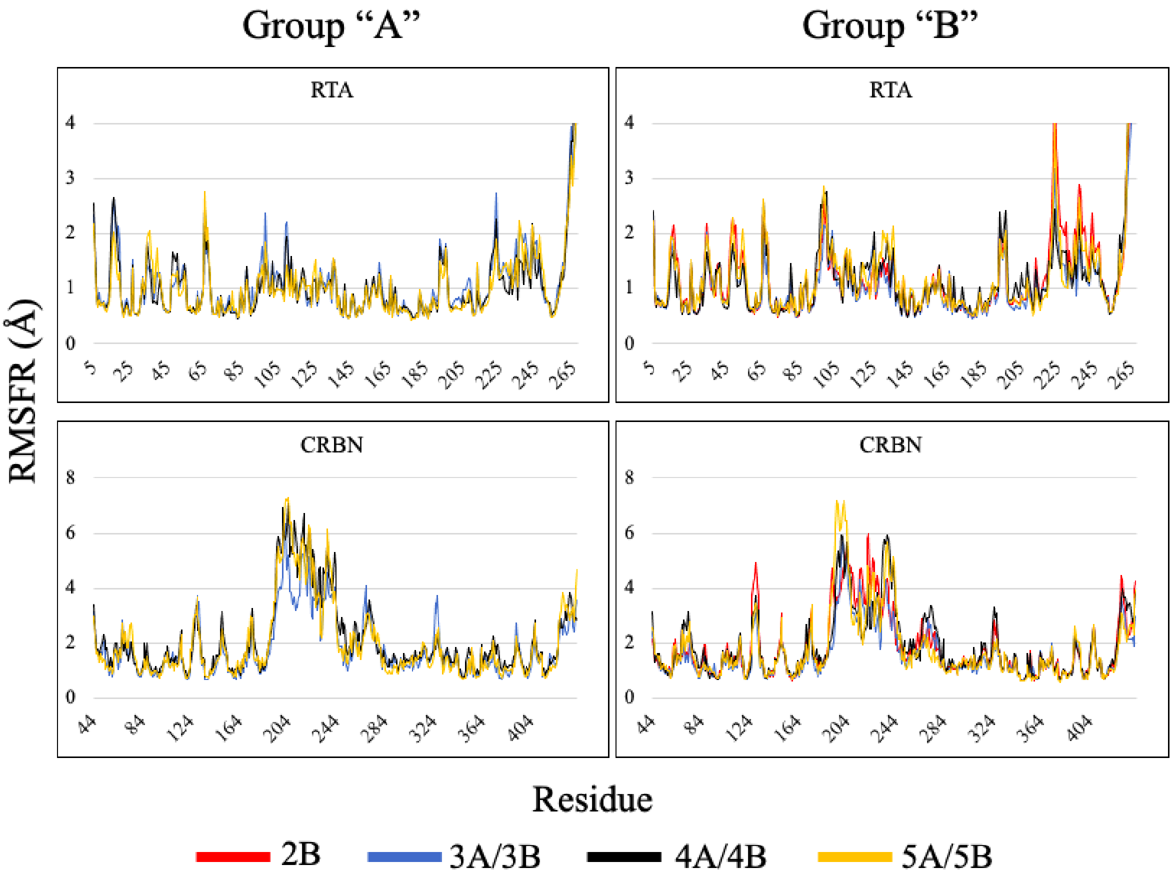

Figure S4. Plots of RMSF per residue for RTA and CRBN over 100 ns MD simulations. Each line represents the mean value among triplicates.

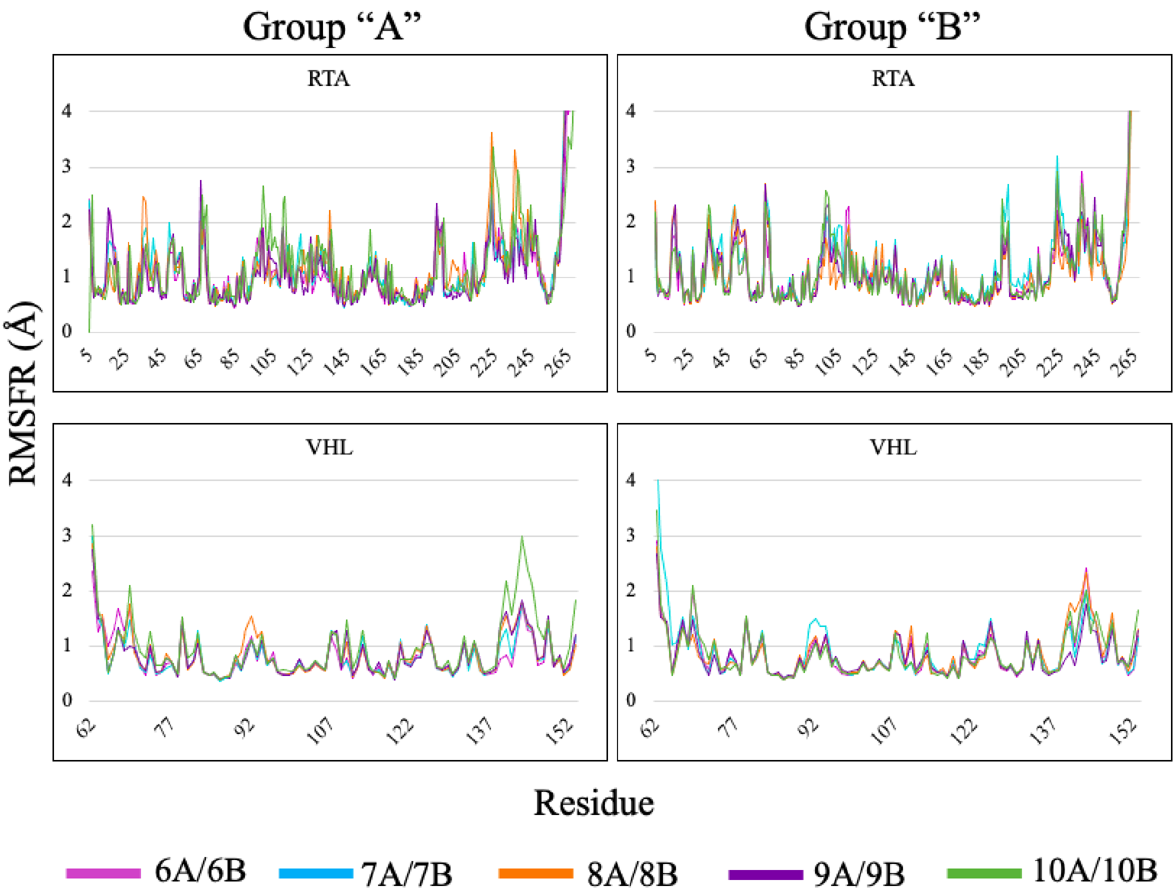

Figure S5. Plots of RMSF per residue for RTA and VHL over 100 ns MD simulations. Each line represents the mean value among triplicates.

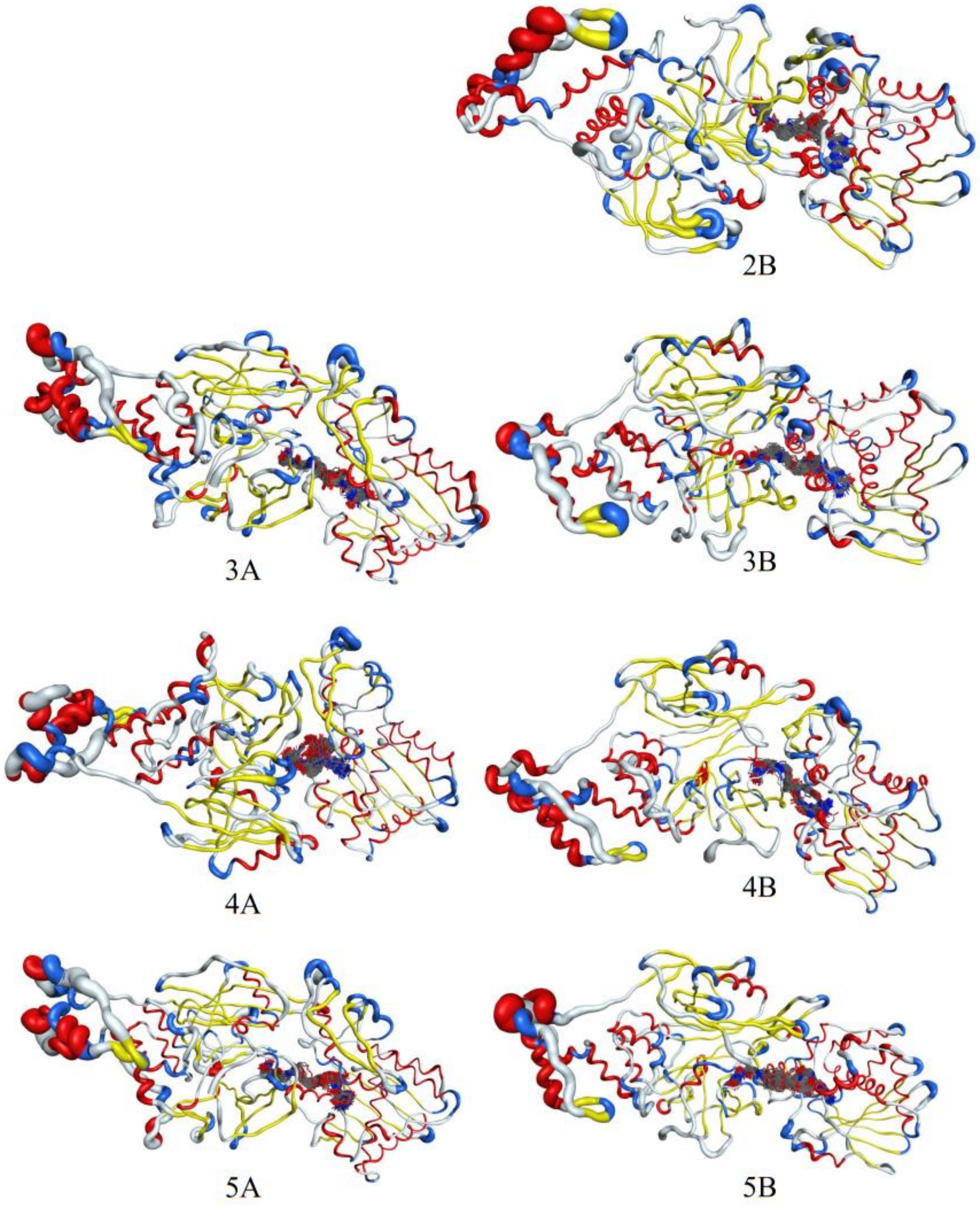

Figure S6. Sausage representation of RTA and CRBN fluctuations over a 100 ns MD simulation, where tube thickness is proportional to residue RMSF values. Secondary structure elements are coloured as follows: α-helices in red, β-sheets in yellow, turns in blue, and loops in light gray. The PROTAC is shown as a superposition of conformations extracted from trajectory frames at 2 ns intervals, illustrating its mobility within the binding site.

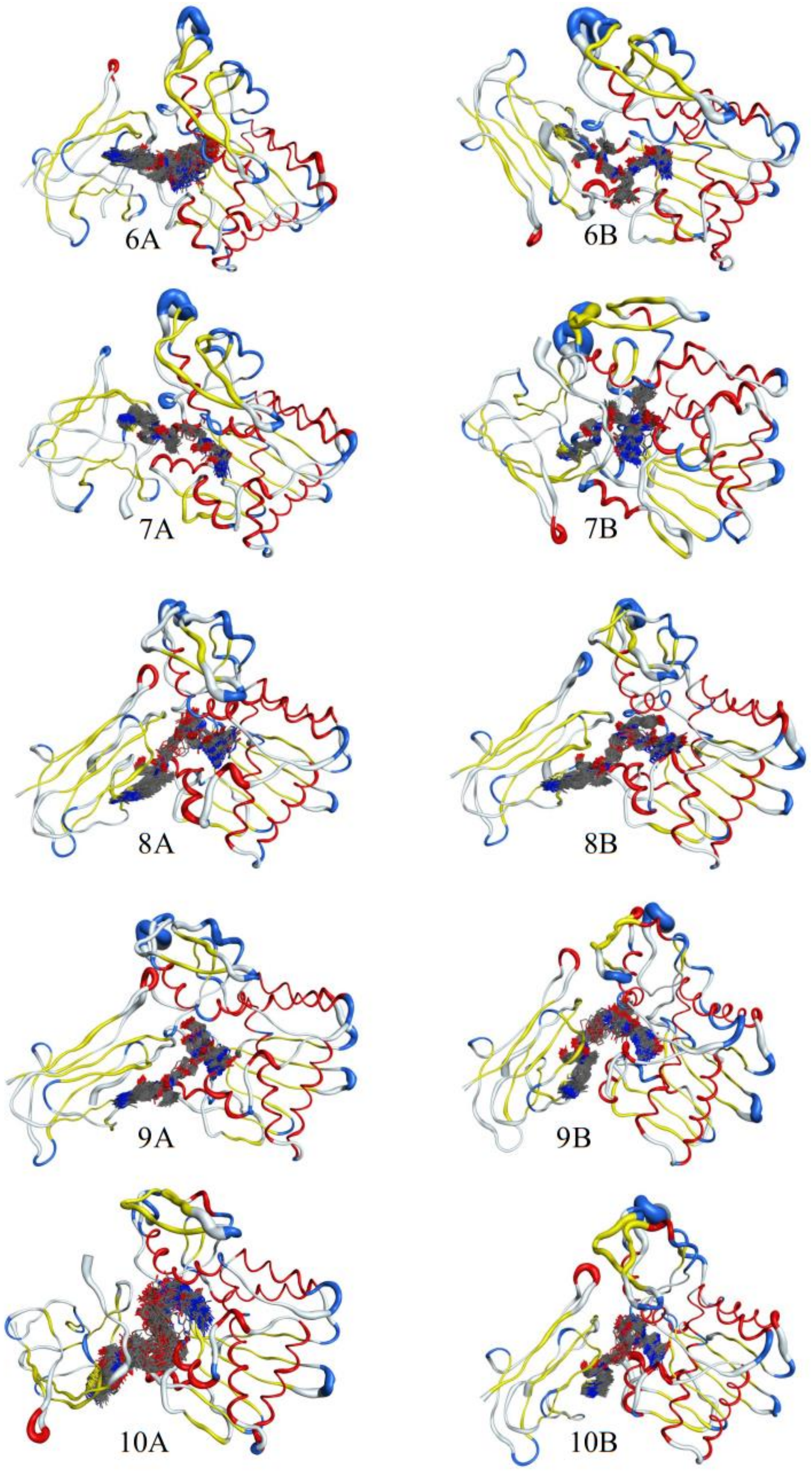

Figure S7. Sausage representation of RTA and VHL fluctuations over a 100 ns MD simulation, where tube thickness is proportional to residue RMSF values. Secondary structure elements are coloured as follows: α-helices in red, β-sheets in yellow, turns in blue, and loops in light gray. The PROTAC is shown as a superposition of conformations extracted from trajectory frames at 2 ns intervals, illustrating its mobility within the binding site.
